# BISON: bi-clustering of spatial omics data with feature selection

**DOI:** 10.1093/bioinformatics/btaf495

**Published:** 2025-09-09

**Authors:** Bencong Zhu, Alberto Cassese, Marina Vannucci, Michele Guindani, Qiwei Li

**Affiliations:** Department of Statistics, The Chinese University of Hong Kong, Shatin, New Territories, Hong Kong; Department of Mathematical Sciences, The University of Texas at Dallas, Richardson, TX 75080, United States; Department of Statistics, Computer Science, Applications “G. Parenti”, University of Florence, Florence 50121, Italy; Department of Statistics, Rice University, Houston, TX 77005, United States; Department of Biostatistics, University of California, Los Angeles, Los Angeles, CA 90024, United States; Department of Mathematical Sciences, The University of Texas at Dallas, Richardson, TX 75080, United States

## Abstract

**Motivation:**

The advent of next-generation sequencing-based spatially resolved transcriptomics (SRT) techniques has reshaped genomic studies by enabling high-throughput gene expression profiling while preserving spatial and morphological context. Understanding gene functions and interactions in different spatial domains is crucial, as it can enhance our comprehension of biological mechanisms, such as cancer-immune interactions and cell differentiation in various regions. It is necessary to cluster tissue regions into distinct spatial domains and identify discriminating genes (DGs) that elucidate the clustering result, referred to as spatial domain-specific DGs. Existing methods for identifying these genes typically rely on a two-stage approach, which can lead to the phenomenon known as *double-dipping*.

**Results:**

To address the challenge, we propose a unified Bayesian latent block model that simultaneously detects a list of DGs contributing to spatial domain identification while clustering these DGs and spatial locations. The efficacy of our proposed method is validated through a series of simulation experiments, and its capability to identify DGs is demonstrated through applications to benchmark SRT datasets.

**Availability and implementation:**

The R/C++ implementation of BISON is available at https://github.com/new-zbc/BISON.

## 1 Introduction

Spatially resolved transcriptomics (SRT) technologies have been rapidly developed and widely used in biomedical research over the past years. These innovative technologies fall into two mainstreams: (i) imaging-based SRT platforms, including seqFISH (Lubeck *et al.* 2014), MERFISH (Chen *et al.* 2015), STARmap (Wang *et al.* 2018), and (2) next-generation sequencing (NGS)-based SRT platforms, such as spatial transcriptomics (ST) (Ståhl *et al.* 2016), 10x Visium ST, high-definition ST (Vickovic *et al.* 2019), Slide-seq (Rodriques *et al.* 2019). The former are typically limited to hundreds of pre-selected genes, whereas the latter can reconstruct a transcriptome-wide spatial map covering expression levels of tens of thousands of protein-coding genes, providing a more comprehensive understanding. With these advancements, NGS-based SRT techniques have become pivotal in discovering novel insights in biomedical research.

The rise of ST has motivated the development of new statistical methods that handle the identification of spatially variable genes (SVGs), i.e. genes with spatial patterns of expression variation across the tissue sample. Recently, Yan *et al.* (2025) have summarized 34 state-of-the-art methods associated with SVG detection, categorizing SVGs into three types: overall, cell type-specific, and spatial domain-marker SVGs. The overall SVGs are defined as the genes that exhibit non-random spatial expression patterns, whose representative detection methods include Trendsceek (Edsgärd *et al.* 2018), spatialDE (Svensson *et al.* 2018), SPARK (Sun *et al.* 2020), BOOST-GP (Li *et al.* 2021), BOOST-MI (Jiang *et al.* 2022), BOOST-HMI (Yang *et al.* 2024), BSP (Wang *et al.* 2023). The cell type-specific SVGs are the genes that exhibit non-random spatial expression patterns within a cell type. The related methods include CTSV (Yu and Luo 2022), C-SIDE (Cable *et al.* 2022), and spVC (Yu and Li 2024) ultilize both SRT data and external cell type annotations. The spatial domain-marker SVGs are defined as the genes that exhibit significantly higher expression in a spatial domain compared to other domains. The methods for detecting spatial domain-marker SVGs first partition the tissue into multiple mutually exclusive spatial domains and then conduct hypothesis tests to evaluate differences in gene mean expression across these spatial domains. For example, SpaGCN (Hu *et al.* 2021) identifies spatial domains using a pre-trained graph convolutional network applied to SRT data and the paired histology image. Then, for each gene, it performs Wilcoxon rank-sum tests on normalized expression levels between each domain and the neighboring spots. DESpace (Cai *et al.* 2024) first implements existing spatial clustering methods, such as BayesSpace (Zhao *et al.* 2021), to identify spatial domains and then uses a generalized linear model based on a Negative Binomial distribution to assess if the spatial domains significantly affect a gene’s expression, similar to iIMPACT (Jiang *et al.* 2024).

Despite the large amount of work aforementioned, challenges remain in detecting SVGs, particularly spatial domain-marker SVGs. Firstly, those heuristic two-step procedures for spatial domain-marker SVGs may accumulate estimation errors at each step, leading to an inflated false positive rate. This issue, known as *double-dipping*, arises when the same dataset is used to define spatial clusters (e.g. cell types) and subsequently test for differential gene expression across those clusters (Neufeld *et al.* 2024, Wang *et al.* 2024). Second, some biologically relevant genes may exhibit high expression only within small regions of interest and can be overlooked by methods that fail to account for such localized expression patterns (Yan *et al.* 2025). To address this, Sottosanti and Risso (2023) proposed SpaRTaCo, a Gaussian process-based latent block model to partition gene expression profiles in SRT data into several blocks, thereby identifying highly expressed marker genes within each spatial domain. However, by relying on all gene features, including non-informative ones that lack heterogeneity across spatial domains, this method could introduce noise and complicate the spatial domain identification process. Recent studies (Li *et al.* 2024, Zhu *et al.* 2025) suggest that eliminating non-informative genes can substantially improve the accuracy of spatial domain identification and enhance downstream biological analyses.

**Figure 1.**
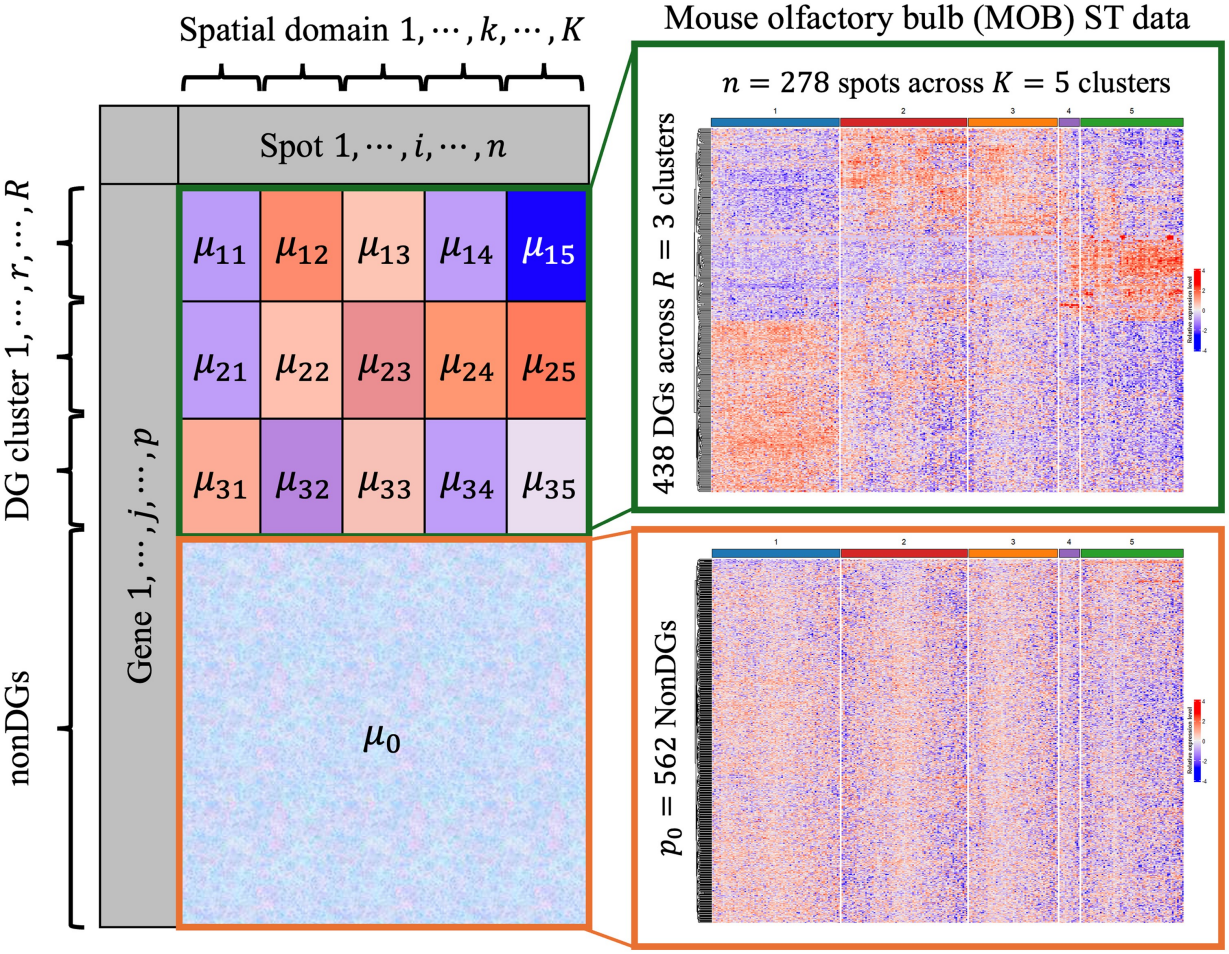
An illustration of the proposed latent block model, BISON, with the null set parameterized by $\mu_{0}$, along with the resulting blocks from the MOB ST data analyzed in Section 4.1.

In response, we develop a unified Bayesian latent block model for bi-clustering of spatial omics data (BISON) with feature selection. BISON simultaneously identifies informative genes that contribute to spatial domain identification and clusters both these genes and spatial spots, as illustrated in Fig. 1. Our key contributions are as follows. First, BISON employs a multivariate count-generating process based on a Poisson model to directly model SRT count data, eliminating the need for *ad hoc* data normalization methods. Second, BISON incorporates a feature selection strategy to generate a list of spatial domain-specific discriminating genes (DGs), enabling a lower-dimensional yet biologically interpretable representation of SRT data. Third, BISON uses a Markov random field (MRF) prior to account for the geospatial structure of SRT data, facilitating the mapping of contiguous domains. Lastly, we introduce a modified integrated complete likelihood criterion to determine the number of gene groups and spatial domains. The effectiveness of BISON is demonstrated through extensive simulation studies. In applications to mouse olfactory bulb (MOB) ST data, human breast cancer (HBC) 10x Visium data, and medial prefrontal cortex (mPFC) STARmap data, BISON outperforms SpaRTaCo and other competing methods. Moreover, the identified DG groups are highly expressed within distinct spatial domains, indicating that each group represents spatial domain-marker genes specific to different spatial domains.

The remainder of the paper is organized as follows: Section 2 presents the detailed formulation of the proposed bi-clustering framework with feature selection, along with posterior inference and model selection. In Sections 3 and 4, we evaluate the proposed model using simulated data and three real SRT datasets from different platforms. Section 5 concludes the paper with a brief discussion about the limitations and potential future avenues.

## 2 Materials and methods

NGS-based SRT platforms, such as ST and the enhanced 10x Visium platforms, measure genome-wide expression levels encompassing over ten thousand genes across thousands of spatial locations referred to as ‘spots’ on a tissue section. The molecular profile is represented by a p×n count matrix $Y_{p\times n}$, with each entry $y_{ji}\in N$ being the read count for gene *j* observed in spot *i*. Here, we use i=1,…,n and j=1,…,p to index spots and genes, respectively. The corresponding geospatial profile is depicted by an n×2 matrix $T_{n\times 2}$, where the *i*th row $t_{i\cdot }=(t_{i1},t_{i2})\in R^{2}$ gives the *x* and *y* coordinates of the *i*th spot’s center. Notably, the *n* spots are approximately arrayed on 2D square or triangular lattices, with each interior spot surrounded by four or six adjacent spots in the ST and 10x Visium platforms, as shown in Supplementary Fig. S10, available as supplementary data at *Bioinformatics* online. This spatial configuration suggests the idea of using a simple spatial dependency structure, which may be sufficient when modeling these data. Indeed, it allows us to alternatively define the geospatial profile by an n×n binary adjacency matrix E, where each entry $e_{ii^{\prime }}=1$ if spot *i* and $i^{\prime }$ are neighbors and $e_{ii^{\prime }}=0$ otherwise. Many Bayesian (see e.g. Zhao *et al.* 2021, Yang *et al.* 2022, Zhu *et al.* 2025) and deep learning-based methods (see e.g. Hu *et al.* 2021, Dong and Zhang 2022) dedicated to spatial domain identification rely on the use of this adjacency matrix. Note that all diagonal entries in E are set to zero by default, i.e. $e_{ii}=0,\forall i$. In the following subsections, we provide the detailed formulation for BISON, with its hierarchical model graphically represented in Supplementary Fig. S1, available as supplementary data at *Bioinformatics* online.

### 2.1 Model formulation

Within DGs, we assume there are *K* spatial domains for spots and *R* gene groups, resulting in R×K latent blocks. We introduce the latent vectors $z={\left(z_{1},\ldots ,z_{n}\right)}^{⊤}$ and $\rho ={\left(\rho_{1},\ldots ,\rho_{p}\right)}^{⊤}$ to denote the latent cluster membership of columns (i.e. spots) and rows (i.e. genes), respectively. Specifically, we define $C_{k}=\{i:z_{i}=k\}$ as the *k*th column cluster and $D_{r}=\{j:\rho_{j}=r\}$ as the *r*th row cluster. The subset $Y^{rk}={\left(y_{ji}\right)}_{j\in D_{r},i\in C_{k}}$ denotes the observations in the *rk*th block. For each entry $y_{ji}\in Y^{rk}$, we model the count using a Poisson distribution as follows:

(1) $$y_{ji}|z_{i}=k,\rho_{j}=r∼\text{Poi}(s_{i}g_{j}\mu_{rk}).$$

We decompose the expected value of the Poisson distribution into a product of the scaling factors $s_{i}\in R^{+}$ and $g_{j}\in R^{+}$, which adjusts for spot-specific effects and gene-specific effects, respectively, and the normalized gene expression level $\mu_{rk}\in R^{+}$. Such a multiplicative formulation of Poisson means is typical in both the frequentist (Witten 2011, Li *et al.* 2012, Cameron and Trivedi 2013) and the Bayesian literature (Banerjee *et al.* 2003, Airoldi and Bischof 2016, Li *et al.* 2017) to accommodate latent heterogeneity and over-dispersion in count data. A simple and practical approach is to set each size factor $s_{i}$ proportional to the total sum of counts in the corresponding spot, i.e. $s_{i}\propto \sum_{j}y_{ij}$ (Sun *et al.* 2020), with the constraint $\sum_{i=1}^{n}s_{i}=1$ to ensure identifiability (Li *et al.* 2021). This leads to $s_{i}=\sum_{j=1}^{p}y_{ji}/(\sum_{i=1}^{n}\sum_{j=1}^{p}y_{ji})$. For the gene-specific effect $g_{j}$, we adopt $g_{j}=\sum_{i}y_{ji}$ as suggested by Witten (2011). In the simulation study of Section 3, we validate the effectiveness of the plug-in estimator by comparing the estimated values ${\hat{s}}_{i}$ and ${\hat{g}}_{j}$ with the true $s_{i}$ and $g_{j}$. As shown in Supplementary Fig. S4, available as supplementary data at *Bioinformatics* online, the estimation values are positively correlated with true values. For the prior of $\mu_{rk}$, we consider the conjugate prior $\mu_{rk}∼\text{Ga}(\alpha_{\mu },\beta_{\mu })$.

As suggested by Zhu *et al.* (2025), numerous non-DGs across spatial domains contribute minimal information for clustering the spots (i.e. columns). Including such non-informative genes may not only complicate the clustering process but also hinder the identification of true column clusters (Tadesse *et al.* 2005). Here, we define non-DG as genes whose expression has no heterogeneity across all spots after adjusting spot and gene-specific effects, implying that the normalized expression level parameters in Equation (1) are constant. To identify non-DGs, we introduce the null gene set $D_{0}=\{j\in \left\{1,\ldots ,p\right\}:\rho_{j}=0\}$, whereby we can consider a total of R+1 gene clusters for the whole set of genes, for notational simplicity. Thus, conditioning on $\rho_{j}=0$, the distribution of observations in the null set $D_{0}$ can be expressed by a Poisson model,

(2) $$y_{ji}|\rho_{j}=0∼\text{Poi}(s_{i}g_{j}\mu_{0}),$$

where we consider the conjugate prior $\mu_{0}∼\text{Ga}(\alpha_{0},\beta_{0})$.

### 2.2 Priors on gene and spot cluster memberships

Let $p_{0}<p$ indicate the total number of non-DGs and, correspondingly, let $p-p_{0}$ be the number of DGs. Following (Sivaganesan *et al.* 2011, Xu *et al.* 2013), we propose a zero-inflated Pólya urn prior for $\rho ={\left(\rho_{1},\ldots ,\rho_{p}\right)}^{⊤}$:

(3) $$P(\rho )=\pi_{0}^{p_{0}}{\left(1-\pi_{0}\right)}^{p-p_{0}}\frac{\gamma^{R}\prod_{r=1}^{R}\Gamma (p_{r})}{\prod_{j=1}^{p-p_{0}}\left(\gamma +j-1\right)},$$

where $p_{r}=|D_{r}|$ for r∈{1,…,R} is the cardinality of gene cluster *r* and γ is the total mass parameter of the Pólya urn scheme. Under this model, $P(\rho_{j}=0)=\pi_{0}$, i.e. gene *j* is a non-DG with probability $\pi_{0}$. When $\rho_{j}\neq 0$, gene *j* is assigned to an existing gene cluster *r* with probability $p_{r}/(p-p_{0})$. Finally, we consider a conjugate Beta prior for $\pi_{0}$ by choosing $\pi_{0}∼\text{Be}(\alpha_{\pi },\beta_{\pi })$.

For the prior distribution of the spot cluster membership vector z, we adopt an MRF model to incorporate available spatial information. In the context of NGS-based SRT data, several statistical models use a similar approach to enhance the spatial coherence of neighboring spots (see e.g. Zhu *et al.* 2018, Liu *et al.* 2022, Jiang *et al.* 2024, Li *et al.* 2024). Under this framework, the conditional distribution of each $z_{i}$ can be expressed as

(4) $$P(z_{i}=k|z_{-i})\propto \;\text{exp}\;\left\{b_{k}+h\sum_{i^{\prime }=1}^{n}e_{ii^{\prime }}I(z_{i^{\prime }}=k)\right\},$$

where $b_{k}$ and *h* are hyperparameters to be chosen and $z_{-i}$ denotes the vector of z excluding the *i*th element. Here $b_{k}$ controls the prior abundance of each cluster, and *h* controls the strength of spatial dependence. We can write the joint MRF prior on z as

(5) $$P(z)\propto \;\text{exp}\;\left\{\sum_{k=1}^{K}b_{k}\sum_{i=1}^{n}I(z_{i}=k)+h\sum_{i<i^{\prime }}e_{ii^{\prime }}I(z_{i}=z_{i^{\prime }})\right\}.$$

For ST and 10x Visium platforms, the binary adjacency matrix E is created based on their square and triangular lattices, respectively. Note that if a spot does not have any neighbors or h=0, its prior distribution reduces to a multinomial prior with parameter $q={\left(q_{1},\ldots ,q_{K}\right)}^{⊤}$ where $q_{k}=\text{exp}(b_{k})/\sum_{k=1}^{K}\;\text{exp}\;(b_{k})$ is a multinomial logistic transformation of $b_{k}$.

### 2.3 Posterior inference

Our study focuses on detecting gene clusters through the gene allocation vector ρ and identifying spatial domains through the cluster allocation vector z. We aim to sample from the posterior distributions of the unknown parameters $\mu =\left\{{\left(\mu_{rk}\right)}_{R\times K},\mu_{0}\right\}$, ρ, and z. The joint posterior can be written as

(6) $$\begin{array}{c} P(\mu ,\rho ,z|Y)\propto P(Y|\mu ,\rho ,z)P(\mu |\rho ,z)P(\rho )P(z)\\ \propto \left\{\prod_{r=1}^{R}\prod_{k=1}^{K}\prod_{i\in C_{k}}\prod_{j\in D_{r}}\text{Poi}(y_{ij}|s_{i}g_{j}\mu_{rk})\text{Ga}(\mu_{rk}|\alpha_{\mu },\beta_{\mu })\right\}\\ \times \left\{\prod_{j\in D_{0}}\prod_{i=1}^{n}\text{Poi}(y_{ij}|s_{i}g_{j}\mu_{0})\text{Ga}(\mu_{0}|\alpha_{0},\beta_{0})\right\}P(z)P(\rho ). \end{array}$$

We developed a Markov Chain Monte Carlo (MCMC) algorithm with a collapsed Gibbs sampler to obtain the posterior samples of ρ and z iteratively by integrating out the model parameter μ. Detailed descriptions of the MCMC procedure are provided in Supplementary Section S1, available as supplementary data at *Bioinformatics* online. Posterior inference on the relevant parameters is achieved *via* post-processing of the MCMC samples after discarding posterior samples in the burn-in period.

A practical challenge related to posterior inference is to summarize a distribution over random partitions. Dahl (2006) addresses the problem by estimating the pairwise probability matrix (PPM). For spots, the ${\text{PPM}}^{\text{spot}}$ is an n×n symmetric matrix, which calculates the posterior pairwise probabilities of co-clustering; that is, the probability that spot *i* and $i^{\prime }$ are assigned to the same cluster: ${\text{PPM}}_{i,i^{\prime }}^{\text{spot}}\approx \sum_{u=1}^{U}I(z_{i}^{\left(u\right)}=z_{i^{\prime }}^{\left(u\right)})/U$, where u=1,…,U represents the iterations after burn-in. A point estimate ${\hat{z}}^{\text{PPM}}$ can then be obtained by obtaining one iteration closest to the PPM:

(7) $${\hat{z}}^{\text{PPM}}=\underset{1\leq u\leq U}{\text{argmin}}\;\sum_{i<i^{\prime }}(I(z_{i}^{\left(u\right)}=z_{i^{\prime }}^{\left(u\right)})-{\text{PPM}}_{ii^{\prime }}^{\text{spot}}{)}^{2}.$$

Similarly, we can also obtain the posterior PPM estimate for ρ. The PPM estimate leverages information from all clusterings through the PPM, providing a comprehensive and representative summary of the clustering results.

### 2.4 Model selection

The number of column clusters *K* and row clusters *R* can be specified based on prior biological knowledge, if available, or, conditionally on the absence of non-DGs, determined using the integrated complete likelihood (ICL) criterion (Biernacki *et al.* 2000). If $p_{0}=0$, the ICL is given by

(8) $$\text{ICL}(R,K)=-\sum_{j=1}^{p}\;\text{log}\;P(y_{j},\hat{z},{\hat{\rho }}_{j};{\left({\hat{\mu }}_{rk}\right)}_{R\times K})+\frac{K-1}{2}\text{log}(n)+\frac{R-1}{2}\text{log}(p)+\frac{KR\nu }{2}\text{log}(np),$$

where ν is the number of parameters per block (Bouveyron *et al.* 2018). In our model, where the non-DGs are estimated, we propose a modified ICL (mICL) criterion that incorporates the contributions of both DGs and non-DGs. Specifically, we consider

(9) $$\begin{array}{c} \text{mICL}(R,K)=-\sum_{j\notin {\hat{D}}_{0}}\;\text{log}\;P(y_{j},\hat{z},{\hat{\rho }}_{j};{\left({\hat{\mu }}_{rk}\right)}_{R\times K})\\ +\frac{K-1}{2}\text{log}(n)+\frac{R-1}{2}\text{log}(p-{\hat{p}}_{0})\\ +\frac{KR\nu }{2}\text{log}\;\{n(p-{\hat{p}}_{0})\}\\ -\sum_{j\in {\hat{D}}_{0}}\;\text{log}\;P(y_{j};{\hat{\mu }}_{0})+\frac{\hat{p_{0}}}{2}\text{log}(n), \end{array}$$

where ${\hat{D}}_{0}=\{j=1,\ldots ,p:{\hat{\rho }}_{j}=0\}$ is the set of estimated non-DGs and ${\hat{p}}_{0}=|{\hat{D}}_{0}|$ is the estimated non-DGs cardinality. The couple (R,K) leading to the lowest mICL value is selected as the most appropriate choice for the data at hand. The details for the computation of mICL are provided in Supplementary Section S1, available as supplementary data at *Bioinformatics* online.

## 3 Results

### 3.1 Simulation study

The spatial pattern in simulations is extracted from the MOB ST data, which contains 278 spots arranged on a square lattice, with the number of spatial domains set to K=4. We evaluate the model’s performance under various settings of the total number of genes and proportion of non-DGs. Specifically, the total number of genes is varied as p∈{500,1000} and the proportion of non-DGs is varied as $\pi_{0}\in \{0,0.2,0.4,0.6,0.8\}$. The number of gene clusters for DGs is R=3, leading μ to be a 3×4 matrix. The observed expression of non-DGs is sampled from a uniform distribution $\mu_{0}∼\text{Unif}(2,6)$. The parameter $\mu_{rk}=4+(k-1)\Delta +(l-1)\Delta$, for k∈{1,…,K} and r∈{1,…,R} with Δ∈{0.5,1,1.5}. The parameter Δ controls the signal of the data generation process. On average, a smaller value of Δ leads to a weaker signal in the dataset. The size factors and gene-specific effects are independent and identically distributed, $s_{i}∼\text{Unif}(0.5,1.5)$ and $g_{j}∼\text{Unif}(0.5,1.5)$. Finally, the count matrix is generated as

$$y_{ij}|\mu_{rk},\mu_{0j},\rho_{j},z_{i}=k∼\{\begin{array}{cc} \text{Poi}\left\{s_{i}g_{j}(\mu_{rk}+\epsilon_{ij})\right\} & \;\text{if}\;\rho_{j}=r\\ \text{Poi}\left\{s_{i}g_{j}(\mu_{0j}+\epsilon_{ij})\right\} & \;\text{if}\;\rho_{j}=0 \end{array},$$

where we add a uniform random noise $\epsilon_{ij}∼\text{Unif}(-0.1,0.1)$ on the mean expression level. In summary, the simulation scheme results in 2×5×3=30 simulation scenarios. For each scenario, we generated 50 replicated datasets.

###  

 We set the hyperparameters of BISON to ensure weakly informative priors. Specifically, the hyperparameters of the Gamma distribution on $\mu_{rk}$ are set as $\alpha_{\mu }=\beta_{\mu }=1$, and similarly, the hyperparameters of the Gamma distribution on $\mu_{0}$ are set as $\alpha_{0}=\beta_{0}=1$. For the Beta distribution governing the probability of a non-DG, we set $\alpha_{\pi }=1$ and $\beta_{\pi }=1$, resulting in a uniform prior with an expected value of 0.5. Regarding the hyperparameters of the MRF prior model, we use $b_{k}=1$ and h=1 following the recommendations of Li *et al.* (2024). The model was run for 10 000 MCMC iterations, with the first 5000 iterations discarded as burn-in, leaving U=5000 posterior samples for analysis.

We compare the performance of BISON in clustering spots and genes against alternative state-of-the-art methods, including SpaRTaCo (Sottosanti and Risso 2023), the bi-clustering algorithm BC and its sparse version sparseBC (with penalty λ=10) in the R package sparseBC (Tan and Witten 2014), and a naive two-directional *K*-means approach, i.e. applying the *K*-means algorithm separately to spots and genes. Implementations of the competing methods are provided in Supplementary Section S2.2, available as supplementary data at *Bioinformatics* online.

To evaluate clustering performance based on spot membership z and gene membership ρ across various methods, we used the adjusted Rand index (ARI, Santos and Embrechts 2009). ARI, which ranges from 0 to 1, measures the similarity between two partitions. We computed ARI using the partition induced by each method under study and the partition used to generate the simulated data. Higher ARI values indicate more accurate clustering performance.

**Figure 2.**
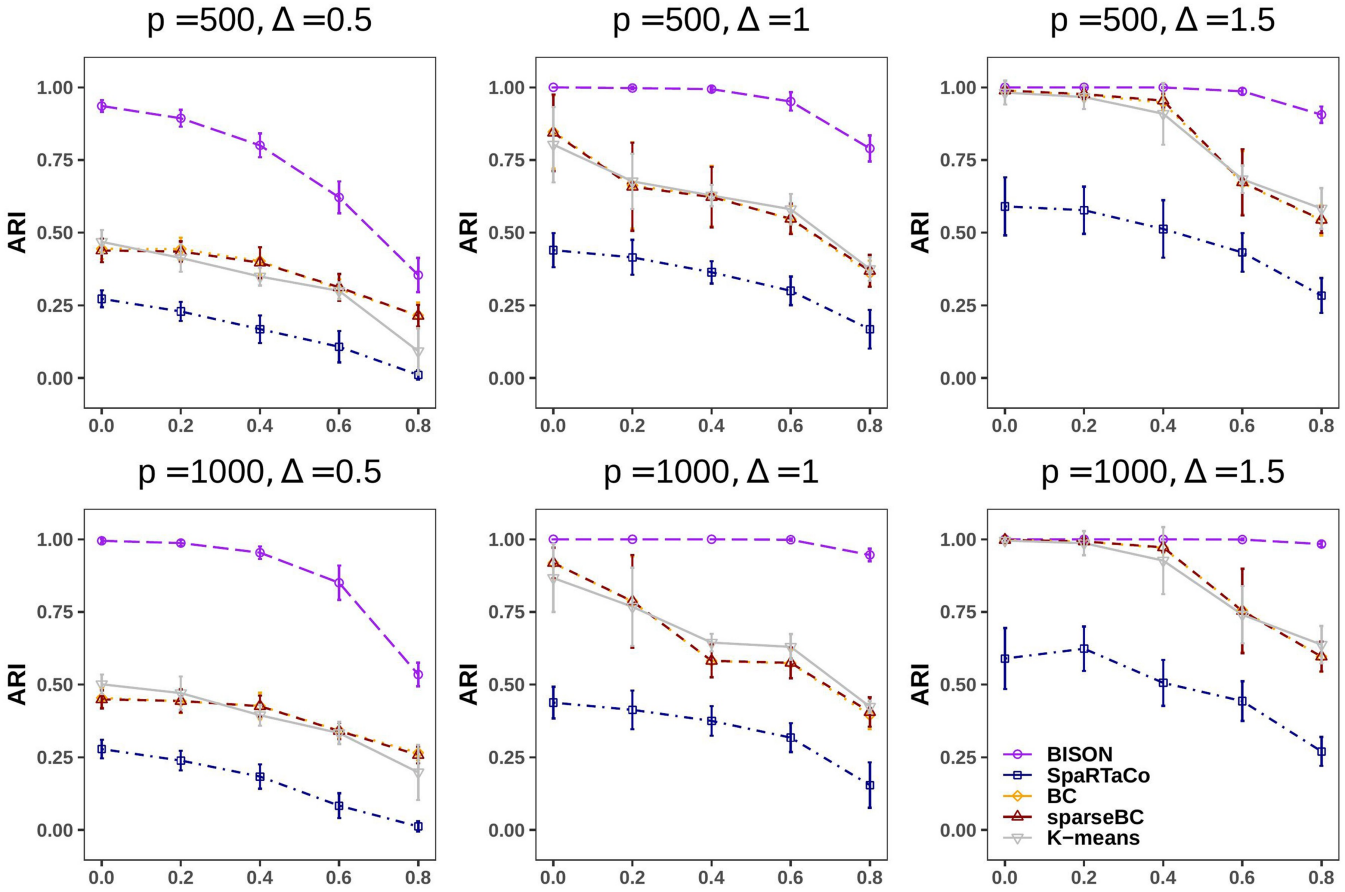
Simulated results: adjust rand index (ARI) for spots clustering against expected proportion of non-DGs $\pi_{0}$. Each subplot represents a specific combination of signal strength (Δ) and the number of genes (*p*), as indicated on top of the subplot. For each scenario, the mean (point) and standard deviation (interval) of the ARI are computed across the 50 generated datasets.

Figure 2 illustrates the spots clustering performance of BISON across the various simulated scenarios, comparing it with the performance of competing methods in terms of ARI for spots clustering. Overall, BISON consistently achieves the highest ARI for spots clustering, indicating superior performance, especially when the signal is weak (Δ = 0.5). The performance of all methods deteriorates as the expected proportion of non-DGs ($\pi_{0}$) increases, suggesting that including non-DGs hinders the performance on spot clustering. Notably, BISON experiences a marked drop in performance when $\pi_{0}$ exceeds 0.6, whereas for the other methods, this decline occurs at lower values of $\pi_{0}$ and is less consistent. Interestingly, SpaRTaCo demonstrates the smoothest decline in performance as $\pi_{0}$ increases. Overall, the performance of the methods has similar patterns when comparing p=500 with p=1000. When $\pi_{0}=0$, the scenario represents the absence of non-DGs. BISON demonstrates superior performance under weak signals and maintains comparable performance under strong signals. This suggests that BISON is more robust compared to the alternative bi-clustering competing methods, in clustering spots. In terms of variability (standard deviation) of the results across the 50 generated datasets per scenario, all the methods seem to have reasonable deviance.

**Figure 3.**
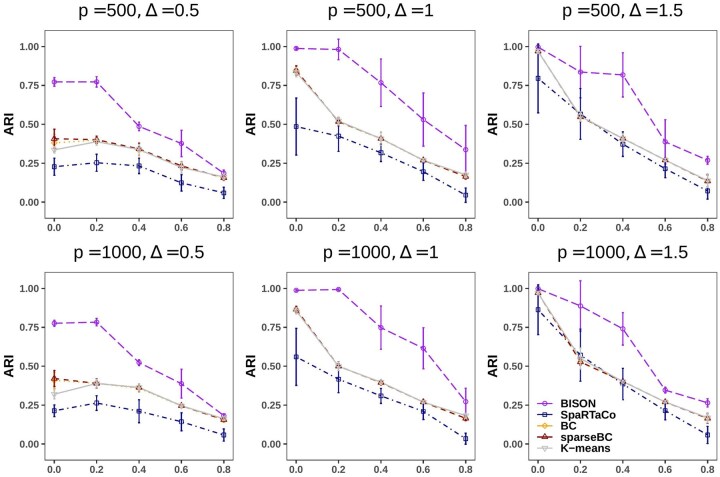
Simulated results: The adjust rand index (ARI) for gene clustering against expected proportion of non-DGs $\pi_{0}$. Each subplot represents a specific combination of signal strength (Δ) and the number of genes (*p*), as indicated on top of the subplot. For each scenario, the mean (point) and standard deviation (interval) of the ARI are computed across the 50 generated datasets.

Figure 3 illustrates the clustering performance of BISON across the various simulated scenarios for gene clustering. Overall, BISON consistently achieves the highest ARI for genes clustering, indicating superior performance. The performance of all methods also deteriorates dramatically as the expected proportion of non-DGs ($\pi_{0}$) increases. This may be attributed to the imbalance between the number of DGs and non-DGs. To further explore this phenomenon, we group all DGs into a single category and evaluate the performance in terms of DG identification. As shown in Supplementary Fig. S5, available as supplementary data at *Bioinformatics* online, the specificity for detecting DGs declines sharply when $\pi_{0}>0.6$, thus this only partially explains the observed results, as in most cases, for most methods, the performance already deteriorates sharply when $\pi_{0}>0.2$. Overall, the performance of the methods has similar patterns when comparing p=500 with p=1000. When comparing the results for different numbers of genes, the patterns and ARI levels are very similar for all methods. When $\pi_{0}=0$, the scenario represents the absence of non-DGs, creating conditions more favorable to the competing models. Nonetheless, BISON demonstrates superior performance under weak signals and maintains comparable performance under strong signals.

Simulation results on clustering performance are based on setting the number of spot and gene clusters in the analysis to match the true values used to generate the simulated data. To further evaluate the mILC criterion’s performance on the simulated datasets, we examined its results for BISON. Supplementary Figs S6 and S7, available as supplementary data at *Bioinformatics* online show the performance in terms of the average estimated number of gene and spot clusters, respectively. Overall, the performance in terms of the number of spot clusters is robust. However, it shows limitations when the number of genes is small (p=500), the signal strength is weak (Δ=0.5), and the expected proportion of non-DGs is high ($\pi_{0}=0.8$). Despite this, such scenarios are unlikely to pose significant issues in real datasets, as one would expect this proportion to be relatively small in many applications. For instance, the estimated proportion of non-DGs is ≈0.1 in the HBC dataset and ≈0.5 in the MOB dataset (see Section 4). As for performance in terms of gene cluster numbers, the results are similar and show only limitations when $\pi_{0}=0.8$.

To evaluate the contribution of including spatial information through the MRF prior and of incorporating feature selection in our model, we conduct an ablation study. Specifically, we run the analysis using two reduced models: one without the MRF prior and the other without feature selection. The performance, in terms of spot clustering and gene clustering, is superior for the complete model, as detailed in Supplementary Section S2.7, available as supplementary data at *Bioinformatics* online. Finally, to investigate the robustness of the methods to model misspecification, we generate simulated data from a Negative Binomial distribution. The Negative Binomial distribution introduces an additional dispersion parameter, $\psi_{j}$, which we simulate as $\psi_{j}∼\;\text{Exp}(0.1)$. Consistent with the results presented earlier in this section, BISON continues to demonstrate the overall best performance, which is detailed in Supplementary Section S2.3, available as supplementary data at *Bioinformatics* online.

##  

### 3.2 Application to the MOB ST data

We consider the MOB ST data, openly accessible through the Spatial Research Lab (https://www.spatialresearch.org/resources-published-datasets/doi-10-1126science-aaf2403/). The preprocessed dataset comprises n=278 spots and p=1000 highly variable genes. To motivate the application of BISON, we first performed an analysis of the MOB ST data where we implemented the clustering with feature selection method BNPSpace of (Zhu *et al.* 2025). This method identifies spatial domains and detects the genes with heterogeneity among domains i.e. DGs. As shown in Fig. 1, this analysis identified 5 domains and 438 DGs, which corresponds to about 44% of the total genes considered. We applied a hierarchical clustering algorithm on the identified DGs, resulting in three distinct gene groups (Fig. 1 and Supplementary Fig. S2, available as supplementary data at *Bioinformatics* online). In contrast, applying the same algorithm to the non-DGs revealed no clear pattern (Fig. 1). These findings illustrate that the expression of identified DGs is heterogeneous across domains, aligning with the definition of DGs. This highlights the need for a framework that can simultaneously discover these gene groups and spatial domains, essentially partitioning the expression matrix into non-overlapping rectangular blocks while excluding non-DGs whose presence could interfere with the accurate identification of the spatial domains.

We then applied BISON to analyze the preprocessed MOB data, using the same prior specifications and algorithm settings as in the simulation study. To determine the optimal number of gene clusters *R* and spot clusters *K*, we computed the mICL for combinations of K∈{2,…,7} and R∈{1,…,7}. Supplementary Table S1, available as supplementary data at *Bioinformatics* online provides the top five mICL values, with the optimal model corresponding to K=4 and R=3. Interestingly, the spot clustering performance, measured by ARI, exhibits a negative correlation with mICL values. This relationship further supports the validity of the proposed mICL criterion, as lower values of mICL correspond to improved clustering results. To assess the MCMC convergence, we ran three independent MCMC chains with diverse initialization under the optimal choice of number of spots and gene clusters. Supplementary Fig. S12, available as supplementary data at *Bioinformatics* online presents the trace plots for these chains, which indicate satisfactory convergence. For posterior inference, we aggregated the outputs from all three chains. For the competing methods, we set the number of gene groups to R+1, allowing one group to capture the non-DG set. The number of spot clusters *K* was kept consistent with the optimal choice used.

**Figure 4.**
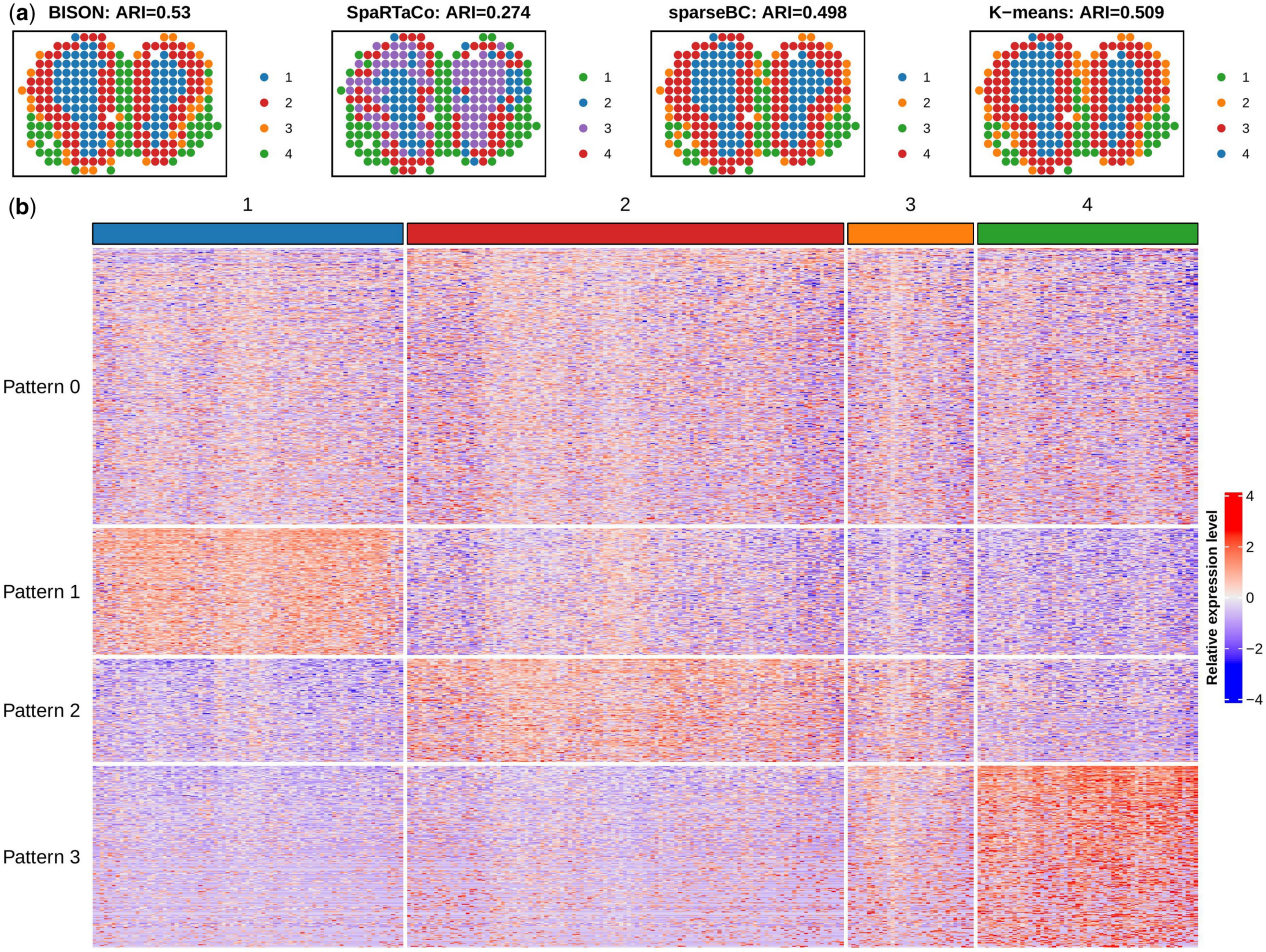
MOB ST data: (a) The spatial domains identified by BISON and competing methods; (b) Heatmap of gene groups identified by BISON. Pattern 0 represents the non-DGs.

**Figure 5.**
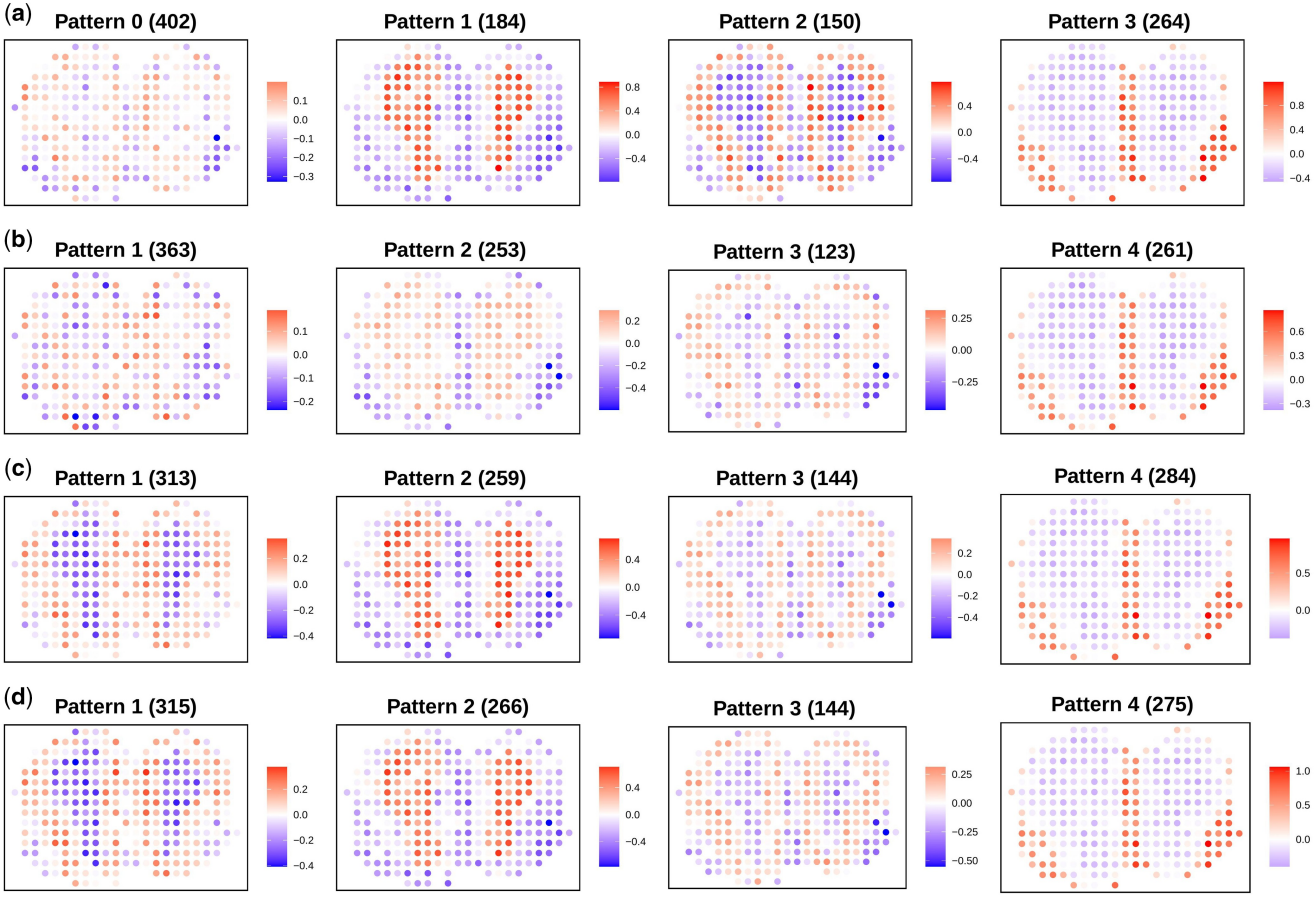
MOB ST data: (a) The spatial expression patterns of gene groups identified by BISON. (b) The spatial expression patterns of gene groups identified by SpaRTaCo. (c) The spatial expression patterns of gene groups identified by sparseBC. (d) The spatial expression patterns of gene groups identified by *K*-means.

The spots of MOB ST data were manually annotated by Ma and Zhou (2022) based on histology and serve as a benchmark to evaluate spot clustering performance. As shown in Fig. 4a, BISON achieved the highest concordance with the manual annotation, with the best ARI =0.561. To evaluate the spatial domain identification performance, we also compared BISON with several representative spatial domain identification methods, including Louvain, BayesSpace (Zhao *et al.* 2021), SpaGCN (Hu *et al.* 2021), and STAGATE (Dong and Zhang 2022). As shown in Supplementary Fig. S15, available as supplementary data at *Bioinformatics* online, BISON achieved similar spatial domain identification performance. The heatmap of gene expression in Fig. 4b displays three DG groups alongside the non-DG group (Pattern 0). Specifically, the 184 genes in the first gene group (Pattern 1) are highly expressed in the first spatial domain, corresponding to the inner layer of the tissue. The 160 genes in the second gene group (Pattern 2) appear to be marker genes for the second and third spatial domains, corresponding to the middle layer of the tissue. Lastly, the 264 genes in the third gene group (Pattern 3) are highly expressed in the last spatial domain, corresponding to the outer layer of the tissue. Figure 5 depicts the spatial expression pattern for each gene group. Genes in Pattern 0 identified by BISON lack spatial information, whereas the genes in the three DG groups exhibit enriched spatial information across distinct spatial regions. Compared to the other bi-clustering methods, BISON discovered the most representative genes for the middle layer. These results align with the findings from the motivating example, further validating the effectiveness of BISON.

### 3.3 Application to the HBC 10x visium data

We applied BISON to analyze an SRT dataset from an HBC study, which includes 2518 spots and 17 651 genes. The dataset is publicly accessible on the 10x Genomics website (https://support.10xgenomics.com/spatial-gene-expression/datasets). The gene expression was measured on a section of a human breast with invasive ductal carcinoma via the 10x Visium platform, along with partially annotated spatial domains from pathologists (Jiang *et al.* 2024). We keep the top p=1000 highly variable genes as the input of our analysis. Similarly as for the analysis of the MOB dataset, we compute the mICL values for combinations of K∈{2,…,7} and R∈{1,…,7}, and found K=5 and R=4 to be the optimal choice (Supplementary Table S2, available as supplementary data at *Bioinformatics* online). We applied BISON, using the same prior specifications and algorithm settings as in the simulation study. Similarly, we ran three independent MCMC chains with diverse initialization under the optimal choice of the number of spot and gene clusters. Supplementary Figure S13, available as supplementary data at *Bioinformatics* online presents the trace plots for these chains, which indicate satisfactory convergence. For posterior inference, we aggregated the outputs from all three chains. For the competing methods, we set the number of gene groups to R+1, allowing one group to capture the non-DG set. The number of spot clusters *K* was kept consistent with the optimal choice used.

**Figure 6.**
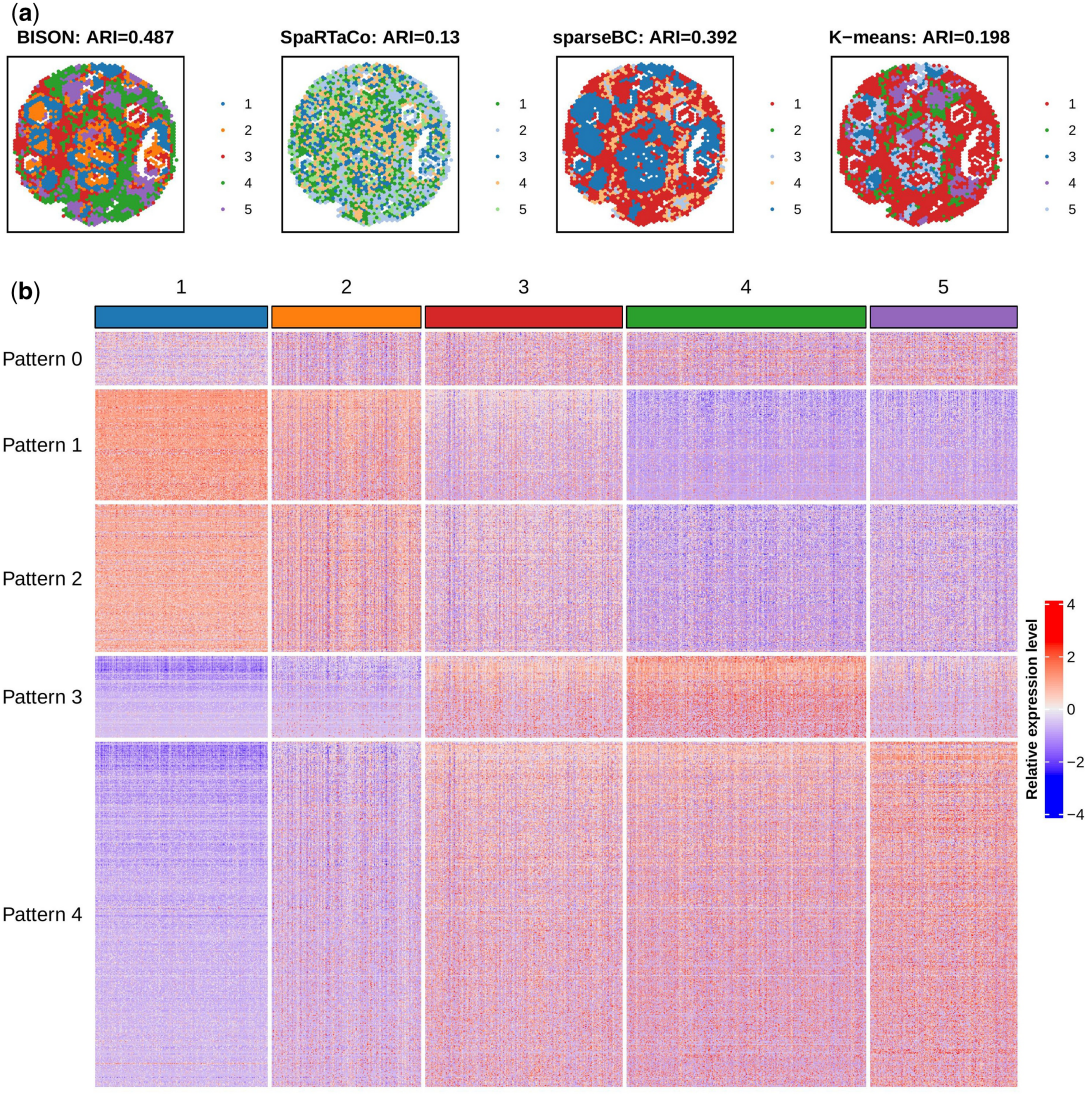
HBC 10x Visium data: (a) The spatial domains identified by BISON and competing methods. (b) Heatmap of gene groups identified by BISON. Pattern 0 represents the non-DGs.

Figure 6a shows the domains detected by the bi-clustering methods, BISON achieved the highest concordance with the manual annotation, with the best ARI =0.487. The comparison with spatial domain identification methods is shown in Supplementary Fig. S16, available as supplementary data at *Bioinformatics* online, where BISON achieved comparable performance. As for the gene groups identified by BISON, as shown in Fig. 6b, the proportion of non-DGs in the HBC data is relatively small (Pattern 0–72 genes, ${\hat{\pi }}_{0}=0.072$). Genes in the first gene group (Pattern 1–150 genes) and in the second gene group (Pattern 2–200 genes) are highly expressed in the first and second spatial domains. Their expression patterns are similar, but they are still identified as separate groups, since the mean expression levels are different, namely ${\hat{\mu }}_{11}/{\hat{\mu }}_{21}=1.59$, and ${\hat{\mu }}_{12}/{\hat{\mu }}_{22}=1.37$. The genes in the third gene group (Pattern 3–108 genes) are highly expressed in the fourth spatial domain and further show a relatively high expression in the third domain. The genes in the fourth gene group (Pattern 4–470 genes) are highly expressed in the fifth spatial domain and further show a relatively high expression in the third and fourth domains. These two gene groups are quite similar to each other, but the third gene group is more representative of the fourth spatial domain, while the fourth gene group is more representative of the fifth spatial domain.

Last, we conducted a gene ontology (GO) enrichment analysis on the identified spatial domain-marker genes using the R package clusterProfiler (Xu *et al.* 2024). We selected annotation terms using a threshold of 0.05 on the adjusted *p*-values (Benjamini and Hochberg 1995). As shown in Supplementary Fig. S18a and b, available as supplementary data at *Bioinformatics* online, genes in Patterns 1 and 2 are related to cell–cell adhesion and cell–substrate adhesion (adjusted *P*-value <.002). Tissue homeostasis is a molecular function that relies on cell–cell adhesion, which is also found to be enriched in our downstream analysis. Molecular mechanisms associated with cell adhesion are frequently linked to diseases ranging from developmental intellectual disability to cancer (Janiszewska *et al.* 2020). The number of significantly enriched biological process GO terms for Patterns 3 and 4 are 59 and 239, respectively. Genes in Pattern 3 play a role in the human immune response, especially in complement activation and B-cell-mediated immunity (see Supplementary Fig. S18c, available as supplementary data at *Bioinformatics* online). Terms related to extracellular organization are found to be enriched in both Patterns 3 and 4, and these are related to proteins regulating tissue structure. As shown in Supplementary Fig. S18d, available as supplementary data at *Bioinformatics* online, the genes in Pattern 4 function in leukocyte migration and proliferation, playing a crucial role in the innate immune system (Janeway *et al.* 2001).

### 3.4 Application to medial prefrontal cortex STARmap data

We applied BISON to an SRT dataset with single-cell resolution from mPFC of the mouse brain (Li and Zhou 2022). This dataset, generated using STARmap technology, contains 1049 cells and 166 genes. The cells in the mPFC were carefully annotated into four distinct layer structures, named L1, L2/3, L5, and L6. These layers consist predominantly of excitatory pyramidal neurons and inhibitory GABAergic interneurons, which orchestrate cortical network dynamics and communicate with long-distance targets (Li and Zhou 2022). We retained all genes as input for our analysis and computed the mICL for combinations of K∈2,…,7 and R∈1,…,7, identifying K=3 and R=3 as the optimal number of cells and DG clusters, respectively (Supplementary Table S3, available as supplementary data at *Bioinformatics* online). Using the same prior specification as in the simulation study, we ran three independent MCMC chains with diverse initializations. The trace plots in Supplementary Fig. S14, available as supplementary data at *Bioinformatics* online indicate satisfactory convergence.

**Figure 7.**
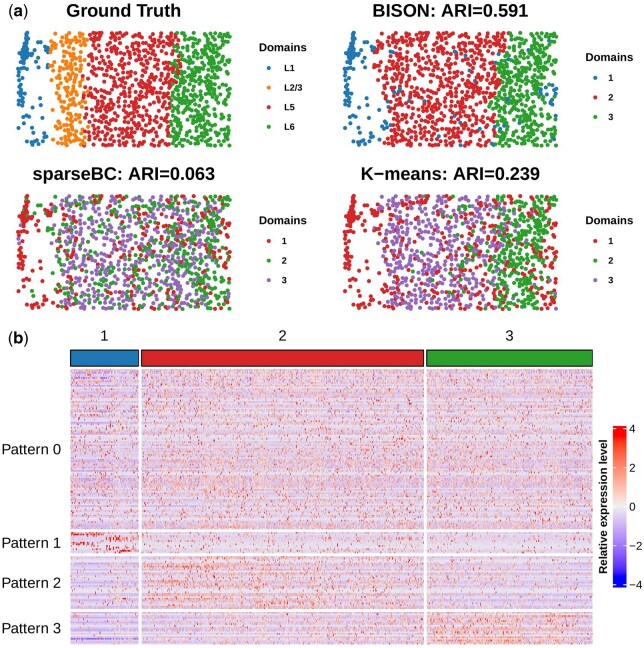
mPFC STARmap data: (a) The spatial domains identified by BISON and competing methods; (b) heatmap of gene groups identified by BISON. Pattern 0 represents the non-DGs.

Figure 7a shows the spatial domains detected by the bi-clustering methods. BISON yielded the most contiguous spatial domains, achieving the highest ARI (=0.591). The competing method, SpaRTaCo, failed to converge when applied to SRT data generated using STARmap technology. Bi-clustering methods that do not incorporate spatial information, including sparseBC and *K*-means, failed to yield contiguous spatial domains. A comparison with spatial domain identification methods is shown in Supplementary Fig. S17, available as supplementary data at *Bioinformatics* online, where BISON achieved the best performance.

Regarding the gene groups identified by BISON, Fig. 7b shows that 100 genes (approximately 10%) were classified as non-DGs (Pattern 0), exhibiting no expression heterogeneity across spatial domains. Thirteen genes (Pattern 1) were identified as markers for spatial domain 1, including known GABAergic interneuron marker genes (Tremblay *et al.* 2016) such as *Gad1*, *Gad2*, *Sst*, and *Vip*. GABAergic neurons are highly heterogeneous and play a critical role in cortical information processing. The number of marker genes for spatial domains 2 (Pattern 2) and 3 (Pattern 3) were 30 and 33, respectively.

To interpret the functional roles of these domains, we conducted a GO enrichment analysis for the genes in Patterns 2 and 3. As shown in Supplementary Fig. S19, available as supplementary data at *Bioinformatics* online, genes in Pattern 2 are involved in cell growth and tissue development, while genes in Pattern 3 are associated with learning, memory, and cognition functions.

## 4 Conclusion

To identify spatial domains and spatial domain-marker genes simultaneously, we have developed a unified Bayesian latent block model, namely BISON. The proposed modeling framework integrates feature selection and clustering of genes through a zero-inflated Pólya urn model, leading to the identification of a list of informative genes that contribute to the spatial domain identification while simultaneously clustering them. To obtain contiguous spatial domains, it efficiently incorporates spatial information and achieves more robust and accurate spatial domains *via* a MRF prior model. Finally, to determine the optimal number of gene groups and spatial domains, we propose a modified Integrated Completed Likelihood criterion. For efficient inference of model parameters, we developed an MCMC algorithm based on a collapsed Gibbs sampler. In our simulation study, BISON achieved superior performance in gene clustering and spatial domain identification under various proportions of non-DGs, different signal-to-noise levels, and gene cardinality. An ablation study showed the importance of including spatial information and of incorporating feature selection. The model also achieved robust performance under model misspecification when simulated data were generated using a Negative Binomial model to produce the final counts. When applied to three real SRT datasets, genes in different DG groups were highly expressed in distinct spatial domains, indicating that the identified gene groups can serve as spatial domain-marker genes. Notably, BISON achieved the highest concordance with the available manual annotations, which serve as the benchmark, when compared to the other bi-clustering methods. In particular, despite our method using a relatively simple spatial dependency structure in the model specification, it achieves a greater ARI than SpaRTaCo, which embeds a Gaussian process. This suggests that employing a simpler structure may be enough for this kind of data. When evaluating spatial domain identification performance, our approach achieved similar or superior results compared to existing methods specifically designed for this task.

Our model presents several limitations that merit future investigation. Firstly, the plug-in estimate of the spot effect $s_{i}$ and gene effect $g_{j}$ may lead to biased estimation of the model parameters. These parameters can be estimated in a unified framework, as suggested by Li *et al.* (2017), albeit this would lead to a higher computational burden. Secondly, the batch effects arising from multi-slice data result in heterogeneity of gene features among batches (Duan *et al.* 2024, Li and Zhang 2024), making it impossible to apply BISON directly to multi-slice SRT data. Thirdly, while the MRF model with the spot adjacency matrix incorporates spatial information locally, it could be enhanced by taking into account the overall locations of the spots, incorporating, for example, a Gaussian process in the model specification. In addition, we proposed a criterion to determine the number of row and column clusters. An elegant alternative, however, would be to model them as random variables, for example, using a Poisson distribution. This approach should be investigated carefully, as posterior inference for these types of models often tends to favor either too many or too few clusters as the dimensionality of the samples increases (Chandra *et al.* 2023).

Finally, another important consideration is computational cost. Among the methods compared in this paper, our approach is the only one based on MCMC simulations, see Supplementary Table S4, available as supplementary data at *Bioinformatics* online. As expected, its computational time is relatively high compared to that of BC, sparseBC, and *K*-means. SpaRTaCo relies on an EM algorithm, and it is also relatively slow. Among all methods, BISON and SpaRTaCo are the least scalable, which is expected given their greater model complexity. For much larger datasets, our method may incur significantly higher computation times. To address this, one could consider implementing a Variational Bayes approach as an alternative to MCMC.

## Supplementary Material

btaf495_Supplementary_Data

## Data Availability

The simulated and real datasets underlying this article are available in our GitHub repository (https://github.com/new-zbc/BISON). The mouse olfactory bulb (MOB) spatial transcriptomics (ST) data were obtained from the public domain through the Spatial Research Lab (https://www.spatialresearch.org/resources-published-datasets/doi-10-1126science-aaf2403/), and the human breast cancer (HBC) 10x Visium data were obtained from the public domain through 10x Genomics (https://support.10xgenomics.com/spatial-gene-expression/datasets).
