# Supplementary material for: BISON: bi-clustering of spatial omics data with feature selection
Source: Bioinformatics. 2025 Sep 9;41(9):btaf495. doi: 10.1093/bioinformatics/btaf495 (PMC12463466; doi:10.1093/bioinformatics/btaf495)
Supplement: btaf495_Supplementary_Data [file btaf495_supplementary_data.pdf]

Supplemental Material for "BISON: Bi-clustering of spatial omics data with feature selection"

Bencong Zhu [1,2], Alberto Cassese[3],
Marina Vannucci[4], Michele Guindani[5], Qiwei Li[2,*]
[1]Department of Statistics,
The Chinese University of Hong Kong, Hong Kong SAR, China
[2]Department of Mathematical Sciences,
The University of Texas at Dallas, Richardson, Texas, U.S.A
[3]Department of Statistics, Computer Science, Applications "G. Parenti",
University of Florence, Florence, Italy
[4]Department of Statistics, Rice University, TX, U.S.A.
[5]Department of Biostatistics,
University of California, Los Angeles, CA, U.S.A.

The supplementary material is organized as follows. Section S1 describes the MCMC algorithm developed for BISON, along with the proposed model selection criterion. Section S2 defines the ARI and other evaluation metrics, details the competing methods, and presents additional results from the simulation study. Finally, Section S3 provides further results from the three case studies.

S1 MCMC algorithms

Following the manuscript, let $\boldsymbol{Y}_{p\times n}$ represent the molecular profile data, where p denotes the number of genes and n the number of spots. Based on our model specification, the MCMC steps are developed for the interaction effect parameters $\boldsymbol{\mu} = \{(\mu_{rk})_{R\times K}, \mu_0\}$, where μ_{rk} represents the cluster interaction effects ($R \times K$), and μ_0 is the baseline characterizing nonDGs. Additionally, MCMC steps are developed for $\boldsymbol{z}$ and $\boldsymbol{\rho}$, which correspond to the spot and gene memberships, respectively. Using this notation, the data likelihood can be factorized as

$$P(\boldsymbol{Y}_{p\times n} \mid \boldsymbol{\mu}, \boldsymbol{\rho}, \boldsymbol{z}, \boldsymbol{s}, \boldsymbol{g}) = \left\{ \prod_{r=1}^{R} \prod_{k=1}^{K} \prod_{\{i:z_i=k\}} \prod_{\{j:\rho_j=r\}} \text{Poi}(y_{ij}|s_i g_j \mu_{rk}) \right\} \times \left\{ \prod_{\{j:\rho_j=0\}} \prod_{i=1}^{n} \text{Poi}(y_{ij}|s_i g_j \mu_0) \right\}, \tag{S1}$$

where the first factor corresponds to the likelihood for the DGs ($\rho_j \neq 0$), and the second factor represents the likelihood for the nonDGs ($\rho_j = 0$).

Spatial information is incorporated through a Markov random field prior on spot membership. An equivalent representation of this prior, as described in the manuscript, is given by

$$P(\boldsymbol{z}) \propto \exp\left\{ \sum_{k=1}^{K} b_k \sum_{i=1}^{n} \text{I}(z_i = k) + h \sum_{i<i'} e_{ii'} \text{I}(z_i = z_{i'}) \right\}. \tag{S2}$$

Here, b_k for $k \in 1, \ldots, K$ represents the prior abundance of each spatial domain. Without spatial information (i.e., $h = 0$), this prior probability simplifies to $P(z_i = k) = \exp(b_k)/\exp\left(\sum_{k'} b_{k'}\right)$, indicating that a higher value of b_k, while keeping the other $b_{k''}$ ($k'' \neq k$) constant, encourages domain k to have a larger cardinality. Since there is no prior information on the abundance of each spatial domain, we set $b_1 = \cdots = b_K = 1$, ensuring that, a priori, each domain is expected to have the same size.

Finally, we remind the reader that a zero-enriched Pólya urn prior is chosen for gene membership. Additionally, a conjugate Gamma prior is applied to the elements of $\boldsymbol{\mu}$, specifically $\mu_{rk} \sim \text{Ga}(\alpha_\mu, \beta_\mu)$ and $\mu_0 \sim \text{Ga}(\alpha_0, \beta_0)$. Consequently, the joint posterior distribution of the model parameters and cluster memberships can be expressed as follows, which is equivalent to the formulation provided in the main manuscript:

$$P((\mu_{rk})_{R\times K}, \mu_0, \boldsymbol{\rho}, \boldsymbol{z}|\boldsymbol{Y}) \propto P(\boldsymbol{Y}|(\mu_{rk})_{R\times K}, \mu_0, \boldsymbol{\rho}, \boldsymbol{z}) P(\boldsymbol{\rho}) P(\boldsymbol{z}) P(\mu_0) P\left((\mu_{rk})_{R\times K}\right). \tag{S3}$$

Figure S1 shows a graphical representation of BISON. To obtain samples of $\boldsymbol{\mu} = \{(\mu_{rk})_{R\times K}, \mu_0\}$, $\boldsymbol{\rho}$, and $\boldsymbol{z}$, we utilized the Gibbs sampler iteratively.

S1.1 Update gene membership $\boldsymbol{\rho}$

For the posterior of $\boldsymbol{\rho}$, we implement the collapsed Gibbs algorithm by integrating out the nuisance parameters $(\mu_{rk})_{R\times K}$ and μ_0. For gene j, the conditional posterior of $\rho_j = 0$ is given by

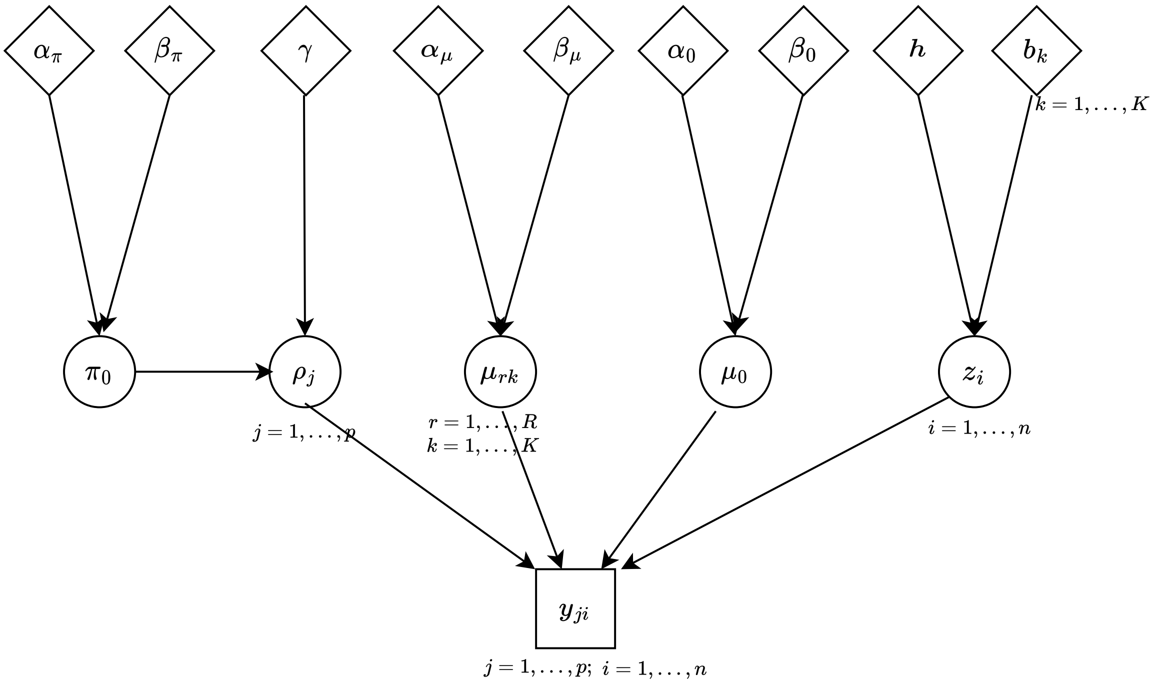

Figure S1: Graphical representation of the proposed BISON model. Observable data, model parameters, and fixed hyperparameters are denoted by square, circle, and diamond-shaped nodes, respectively. A link between two nodes represents a direct probabilistic dependence.

$$
\begin{aligned}
&P(\rho_j = 0 | \boldsymbol{\rho}_{-j}, \boldsymbol{z}, \boldsymbol{Y}) \\
&\propto \pi_0 \int \prod_{i=1}^{n} \text{Poi}(y_{ji} | s_i g_j \mu_0) p(\mu_0 | \boldsymbol{\rho}_{-j}, \boldsymbol{z}, \boldsymbol{Y}_{-j}) d\mu_0 \\
&\propto \pi_0 \int \prod_{i=1}^{n} \frac{(s_i g_j)^{y_{ji}}}{y_{ji}!} \frac{\beta_{old}^{\alpha_{old}}}{\Gamma(\alpha_{old})} \mu_0^{\alpha_{old} + y_{ji} - 1} \exp[-(\beta_{old} + s_i g_j)\mu_0] d\mu_0 \\
&\propto \pi_0 \prod_{i=1}^{n} \frac{(s_i g_j)^{y_{ji}}}{y_{ji}!} \frac{\beta_{old}^{\alpha_{old}}}{\Gamma(\alpha_{old})} \frac{\Gamma(\alpha_{new})}{\beta_{new}^{\alpha_{new}}},
\end{aligned}
$$

where $\alpha_{old} = \alpha_0 + \sum_{i=1}^{n} \sum_{\{j':\rho_{j'}=0, j' \neq j\}} y_{j'i}$ and $\beta_{old} = \beta_0 + \sum_{i=1}^{n} \sum_{\{j':\rho_{j'}=0, j' \neq j\}} s_i g_{j'}$, and the new values are obtained as $\alpha_{new} = \alpha_0 + \sum_{i=1}^{n} \sum_{\{j':\rho_{j'}=0\}} y_{j'i}$ and $\beta_{new} = \beta_0 + \sum_{i=1}^{n} \sum_{\{j':\rho_{j'}=0\}} s_i g_{j'}$. Similarly, for gene j the conditional posterior of $\rho_j = r$ is given by

$$
\begin{aligned}
& P(\rho_j = r | \boldsymbol{\rho}_{-j}, \boldsymbol{z}, \boldsymbol{Y}) \\
\propto\ & P(\boldsymbol{Y}_{j\cdot} | \rho_j = r, \boldsymbol{\rho}_{-j}, \boldsymbol{z}, \boldsymbol{Y}_{-j}) P(\rho_j = r | \boldsymbol{\rho}_{-j}) \\
\propto\ & (1-\pi_0) \frac{p_{r,-j}}{p - p_0 - 1} \prod_{k=1}^{K} \int \prod_{i \in \mathcal{C}_k} \mathrm{Poi}(y_{ji} | s_i g_j \mu_{rk}) p(\mu_{rk} | \boldsymbol{\rho}_{-j}, \boldsymbol{z}, \boldsymbol{Y}_{-j}) d\mu_{rk} \\
\propto\ & (1-\pi_0) \frac{p_{r,-j}}{p - p_0 - 1} \prod_{i=1}^{n} \frac{(s_i g_j)^{y_{ji}}}{y_{ji}!} \prod_{k=1}^{K} \frac{\beta_{k,old}^{\alpha_{k,old}}}{\Gamma(\alpha_{k,old})} \frac{\Gamma(\alpha_{k,new})}{\beta_{k,new}^{\alpha_{k,new}}},
\end{aligned}
$$

where $p_{r,-j} = |\{j' \neq j : \rho_{j'} = r\}|$. The other values $\alpha_{k,old} = \alpha_\mu + \sum_{i \in \mathcal{C}_k} \sum_{\{j' : \rho_{j'} = r, j' \neq j\}} y_{j'i}$ and $\beta_{k,old} = \beta_\mu + \sum_{i \in \mathcal{C}_k} \sum_{\{j' : \rho_{j'} = r, j' \neq j\}} s_i g_{j'}$, and the new values are obtained as $\alpha_{k,new} = \alpha_\mu + \sum_{i \in \mathcal{C}_k} \sum_{\{j' : \rho_{j'} = r\}} y_{j'i}$ and $\beta_{k,new} = \beta_\mu + \sum_{i \in \mathcal{C}_k} \sum_{\{j' : \rho_{j'} = r\}} s_i g_{j'}$. Next, we normalize $P(\rho_j = r \mid \cdot)$ for $r = 0, \ldots, R$, to obtain the cluster membership probabilities as $p_r = \frac{P(\rho_j = r|\cdot)}{\sum_{r'=0}^{R} P(\rho_j = r'|\cdot)}$. We then sample ρ_j from the categorical distribution $\rho_j \sim \mathrm{Mult}(1, \boldsymbol{p}^*)$, where $\boldsymbol{p}^* = (p_0, \ldots, p_R)^\top$ and the support is $0, 1, \ldots, R$.

S1.2 Update spot membership $\boldsymbol{z}$

For the posterior of $\boldsymbol{\rho}$, we implement the collapsed Gibbs algorithm by integrating out the nuisance parameters $(\mu_{rk})_{R\times K}$ and μ_0. For spot i, the conditional posterior of $z_i = k$ is given by

$$
\begin{aligned}
& P(z_i = k|\boldsymbol{z}_{-i}, \boldsymbol{\rho}, \boldsymbol{Y}) \\
\propto\ & P(\boldsymbol{Y}_i|z_i = k, \boldsymbol{z}_{-i}, \boldsymbol{\rho}, \boldsymbol{Y}_{-i})P(z_i = k|\boldsymbol{z}_{-i}) \\
\propto\ & \exp[b_k n_{k,-i} + h\sum_{i'\neq i} e_{ii'} I(z_{i'} = k)] \prod_{r=1}^{R} \int \prod_{j\in\mathcal{D}_r} \mathrm{Poi}(y_{ji}|s_i g_j \mu_{rk}) p(\mu_{rk}|\boldsymbol{\rho}_{-j}, \boldsymbol{z}, \boldsymbol{Y}) d\mu_{rk} \\
\propto\ & \exp[b_k n_{k,-i} + h\sum_{i'\neq i} e_{ii'} I(z_{i'} = k)] \prod_{r=1}^{R} \frac{\beta_{r,old}^{\alpha_{r,old}}}{\Gamma(\alpha_{r,old})} \frac{\Gamma(\alpha_{r,new})}{\beta_{r,new}^{\alpha_{r,new}}},
\end{aligned}
$$

where $\alpha_{r,old} = \alpha_\mu + \sum_{j\in\mathcal{D}_r} \sum_{\{i':z'_i=k,i'\neq i\}} y_{ji'}$ and $\beta_{r,old} = \beta_\mu + \sum_{j\in\mathcal{D}_r} \sum_{\{i':z'_i=k,i'\neq i\}} s_{i'} g_j$, and the new values are obtained as $\alpha_{r,new} = \alpha_\mu + \sum_{j\in\mathcal{D}_r} \sum_{\{i':z'_i=k\}} y_{ji'}$ and $\beta_{r,new} = \beta_\mu + \sum_{j\in\mathcal{D}_r} \sum_{\{i':z'_i=k\}} s_{i'} g_j$. Next, we normalize $P(z_i = k|\cdot)$ for $k = 1, \ldots, K$, to obtain the cluster membership probabilities as $p_k = \frac{P(z_i=k|\cdot)}{\sum_{k'=1}^{K} P(z_i=k'|\cdot)}$. We then sample z_i from the categorical distribution $z_i \sim \mathrm{Mult}(1, \boldsymbol{p}^*)$, where $\boldsymbol{p}^* = (p_1, \ldots, p_K)^\top$ and the support is $\{1, 2, \ldots, K\}$.

S1.3 Update of $\boldsymbol{\mu}$ and $\boldsymbol{\pi_0}$

- Update μ_0 sampling from its posterior distribution,

$$\mathrm{Ga}\left(\sum_{i=1}^{n}\sum_{\{j:\rho_j=0\}} y_{ji}+\alpha_0, \sum_{i=1}^{n}\sum_{\{j:\rho_j=0\}} s_j g_j+\beta_0\right).$$

- For $r = 1, \ldots R$ and $k = 1, \ldots, K$, update μ_{rk} sampling from its posterior distribution, $\mathrm{Ga}\left(\sum_{i\in\mathcal{C}_k}\sum_{j\in\mathcal{D}_r} y_{ji}+\alpha_\mu, \sum_{i\in\mathcal{C}_k}\sum_{j\in\mathcal{D}_r} s_j g_j+\beta_\mu\right)$.

- Update π_0 sampling from its posterior distribution, $\pi_0 \sim \mathrm{Be}(\alpha_\pi + p_0, \beta_\pi + p - p_0)$, where $p_0 = |\mathcal{D}_0|$.

S1.4 Model selection

In the mICL criterion proposed in the manuscript, the log-likelihood term, i.e., the first term in the Equation, is computed as

$$\begin{aligned}\sum_{j\notin\hat{\mathcal{D}}_0}\log P(\boldsymbol{y}_j,\hat{\boldsymbol{z}},\hat{\rho}_j;(\hat{\mu}_{rk})_{R\times K}) &= \sum_{k=1}^{K} n_k\log(a_k)+\sum_{r=1}^{R} p_r\log(b_k)\\ &+\sum_{k=1}^{K}\sum_{r=1}^{R}\sum_{i\in\hat{\mathcal{C}}_k}\sum_{j\in\hat{\mathcal{D}}_r}(\log\frac{(s_i g_j\hat{\mu}_{\hat{\rho}_j\hat{z}_i})^{y_{ji}}}{y_{ji}!}-s_i g_j\hat{\mu}_{\hat{\rho}_j\hat{z}_i})\end{aligned}$$

where $n_k = |\hat{\mathcal{C}}_k|$ is the cardinality of the estimated spot cluster $\hat{\mathcal{C}}_k$, $a_k = n_k/n$ is the proportion of spots assigned to spot cluster k for $k = 1,\ldots,K$, $p_r = |\hat{\mathcal{D}}_r|$ is the cardinality of the estimated gene cluster $\hat{\mathcal{D}}_r$, and $b_r = p_r/(p-\hat{p}_0)$ is the proportion of DGs assigned to cluster r for $r = 1,\ldots,R$.

S2 Simulation study

S2.1 Evaluation metrics

For spot and gene clustering results, we quantified the performance using the Adjusted Rand Index (ARI) (Hubert & Arabie 1985), a variant of the Rand Index (RI) based on the estimation of cluster assignments. We use $\hat{\boldsymbol{z}} = (\hat{z}_1, \ldots, \hat{z}_n)^\top$ as an illustrative example. Define the following: $A = \sum_{i>i'} I(z_i = z_{i'})I(\hat{z}_i = \hat{z}_{i'})$, the number of pairs belonging to the same group in both the true and estimated partitions; $B = \sum_{i>i'} I(z_i = z_{i'})I(\hat{z}_i \neq \hat{z}_{i'})$, the number of pairs belonging to the same group in the true partition but to different groups in the estimated partition; $C = \sum_{i>i'} I(z_i \neq z_{i'})I(\hat{z}_i = \hat{z}_{i'})$, the number of pairs belonging to different groups in the true partition but assigned to the same group in the estimated partition; $D = \sum_{i>i'} I(z_i \neq z_{i'})I(\hat{z}_i \neq \hat{z}_{i'})$, the number of pairs assigned to different groups in both the true and estimated partitions. The ARI is then defined as

$$\text{ARI} = \frac{\binom{n}{2}(A+D) - [(A+B)(A+C) + (C+D)(B+D)]}{\binom{n}{2}^2 - [(A+B)(A+C) + (C+D)(B+D)]}.$$

If we regard the discriminating genes as a single group ($\rho_j \neq 0$), we can evaluate the performance of discriminating gene identification. The metrics sensitivity, specificity, and accuracy can be calculated as

$$\text{Sensitivity} = \frac{\text{TP}}{\text{TP} + \text{FN}},$$
$$\text{Specificity} = \frac{\text{TN}}{\text{TN} + \text{FP}},$$
$$\text{Accuracy} = \frac{TP + TN}{TP + TN + FP + FN}.$$

with TP, TN, FP, and FN denoting the true positives, true negatives, false positives, and false negatives, respectively.

S2.2 Details of competing methods

- **SpaRTaCo**: A co-clustering framework for SRT data based on Gaussian process (Sottosanti & Risso 2023). The R code and tutorial are publicly available at `https://github.com/andreasottosanti/spartaco`. The augments in the main function `spartaco()` include p-by-n log-normalized gene expression profile (x), n-by-2 spatial location matrix (coordinates), and the number of gene clusters (K) and spot clusters (R).

- **sparseBC**: A frequentist bi-clustering approach based on the latent block model, which is publicly available in the R package `sparseBC`. The augments in the main function `sparseBC()` include p-by-n log-normalized gene expression profile (x), the number of gene clusters (k), the number of spot clusters (r), and the penalty (λ).

- **BC**: BC use the same main function `sparseBC()` as **sparseBC**, with the penalty parameter $\lambda = 0$.

- **K-means**: Utilized as the naive baseline method. We use the `kmeans()` function for genes (rows) and spots (columns) separately.

S2.3 Model misspecification

We evaluate the robustness of the model to misspecification by generating simulated data as described in Section 3.1 of the manuscript. However, the final entries of the count matrix $\boldsymbol{Y}_{p\times n}$ are generated via a Negative Binomial distribution. Specifically,

$$y_{ji}|\mu_{rk}, \mu_0, \rho_j, z_i = k \sim \begin{cases} \text{NB}(s_i g_j \mu_{rk}, \psi_j) & \text{if } \rho_j = r \\ \text{NB}(s_i g_j \mu_0, \psi_j) & \text{if } \rho_j = 0 \end{cases},$$

where the dispersion parameter $\psi_j \sim \text{Exp}(0.1)$. Figures S2 and S3 illustrate the spots/genes clustering performance of BISON across the various simulated scenarios, comparing it with the performance of competing methods in terms of ARI for spots/genes clustering. Each subplot represents a specific combination of signal strength (Δ) and the number of genes (p). The x-axis denotes the expected proportion of nonDGs (π_0), while the different lines correspond to the methods being compared. For each scenario, the mean (point) and standard deviation (interval) of the ARI, computed across the 50 generated datasets, are provided. BISON demonstrates the best overall performance, achieving the highest ARI score, especially under the scenarios with weak signal ($\Delta = 0.5$). These results demonstrate the robustness of BISON to model misspecification.

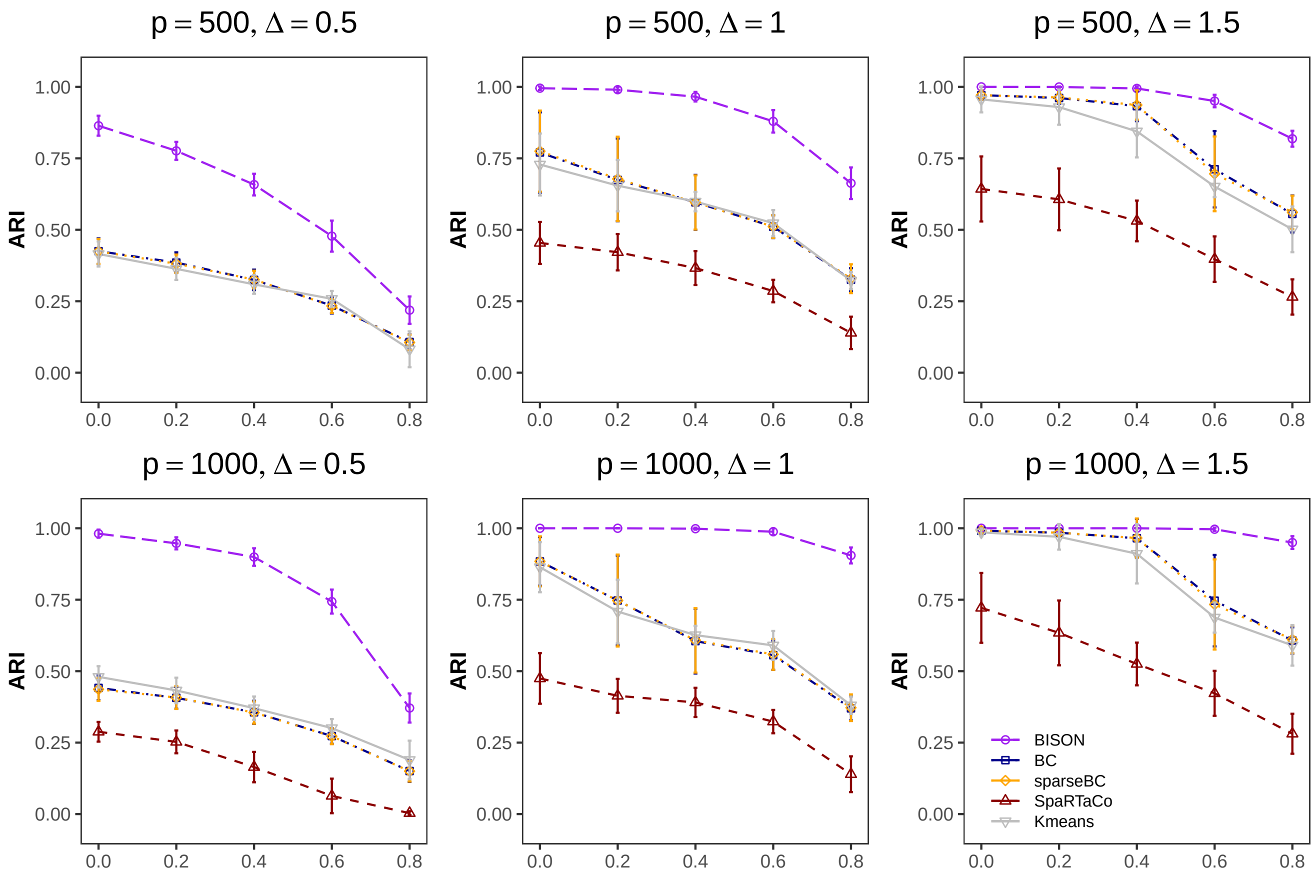

Figure S2: Simulation study under NB generation: ARI for spots clustering against the expected proportion of nonDGs (π_0). Each subplot represents a specific combination of signal strength (Δ) and the number of genes (p), as indicated on top of the subplot. For each scenario, the mean (point) and standard deviation (interval) of the ARI, computed across the 50 generated datasets, are provided.

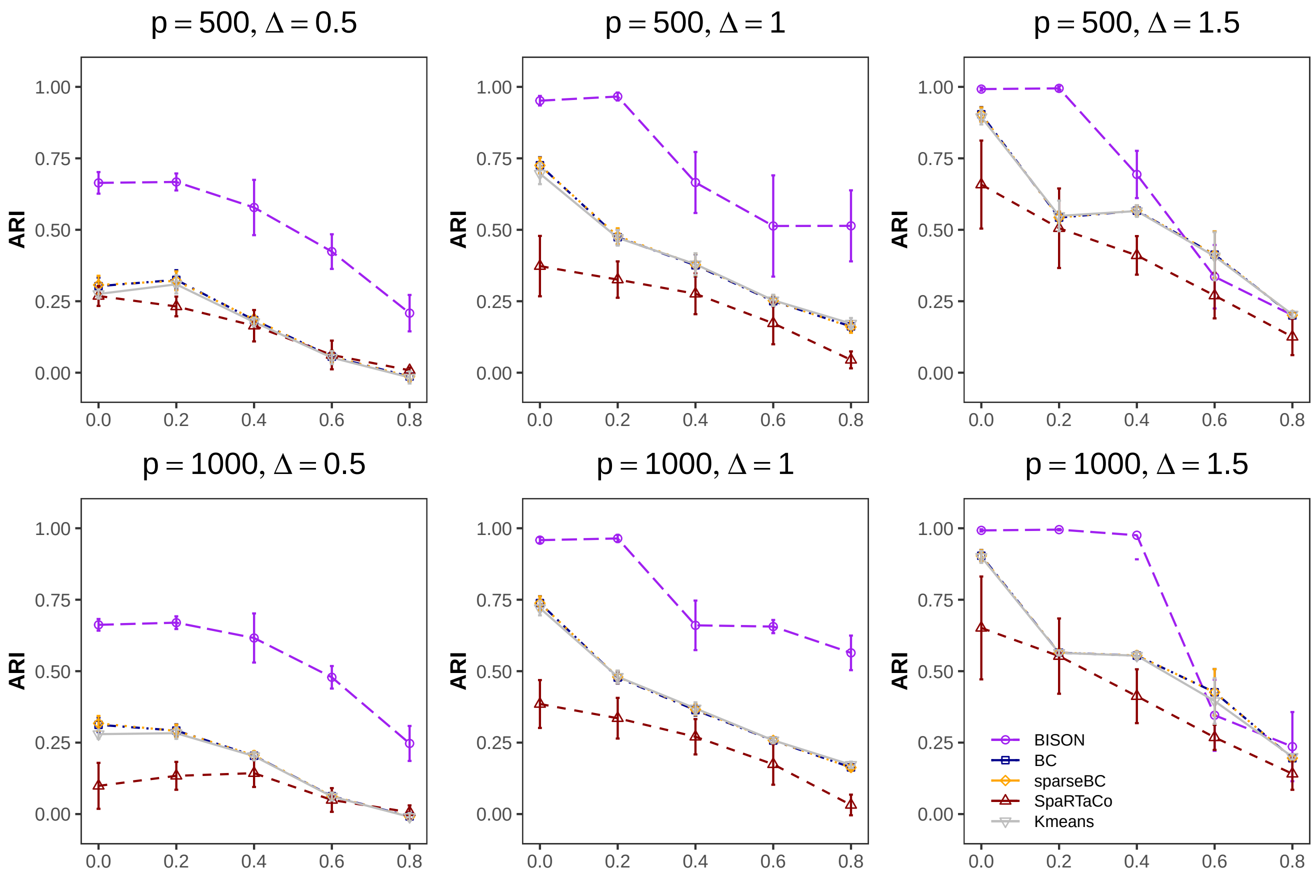

Figure S3: Simulation study under NB generation: ARI for spots clustering against expected proportion of nonDGs (π_0). Each subplot represents a specific combination of signal strength (Δ) and the number of genes (p), as indicated on top of the subplot. For each scenario, the mean (point) and standard deviation (interval) of the ARI, computed across the 50 generated datasets, are provided.

S2.4 Plug-in estimators

Figure S4 shows a scatter plot comparing the estimated values for the production of size factors and gene factors $\hat{s}_i\hat{g}_j$, with their true values $s_i g_j$, in the simulated data under varying proportions of nonDGs, with $p = 500$ and $\Delta = 0.5$. The positive correlation between the estimated and true values suggests that the plug-in estimator provides a valid approximation for $s_i g_j$.

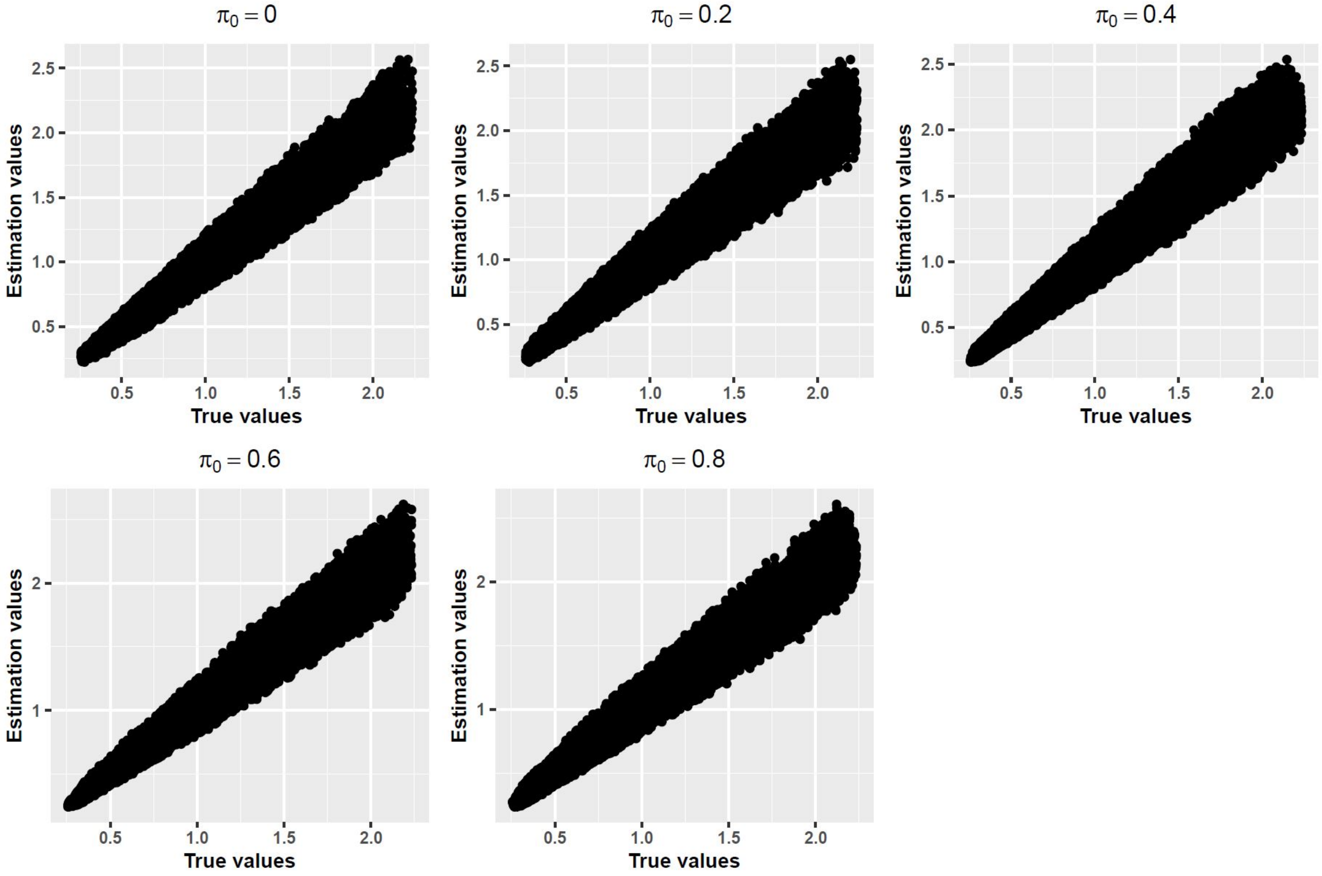

Figure S4: Simulation study: Scatter plot comparing the estimated values for the production of size factors and gene factors, $\hat{s}_i\hat{g}_j$, with their true values, $s_i g_j$, in the simulated data under varying proportions of nonDGs, with $p = 500$ and $\Delta = 0.5$.

S2.5 Discriminating genes selection

Figure S5 illustrates the DGs selection performance of BISON across the various simulated scenarios, in terms of different metrics. Each subplot represents a specific combination of signal strength (Δ) and the number of genes (p). The x-axis denotes the expected proportion of nonDGs (π_0), while the different lines correspond to the different metrics, specifically sensitivity (red), specificity (green) and accuracy (blue). For each scenario, the mean (point) and standard deviation (interval) of each metric, computed across the 50 generated datasets, are provided. Overall, the method appears to perform well in terms of sensitivity. The number of genes seems to have minimal impact, as the upper and lower halves of the matrix of subplots show similar results. However, performance deteriorates across all metrics as the expected proportion of nonDGs increases. Interestingly, sensitivity decreases as the signal strength increases, whereas the other two metrics improve with stronger signals.

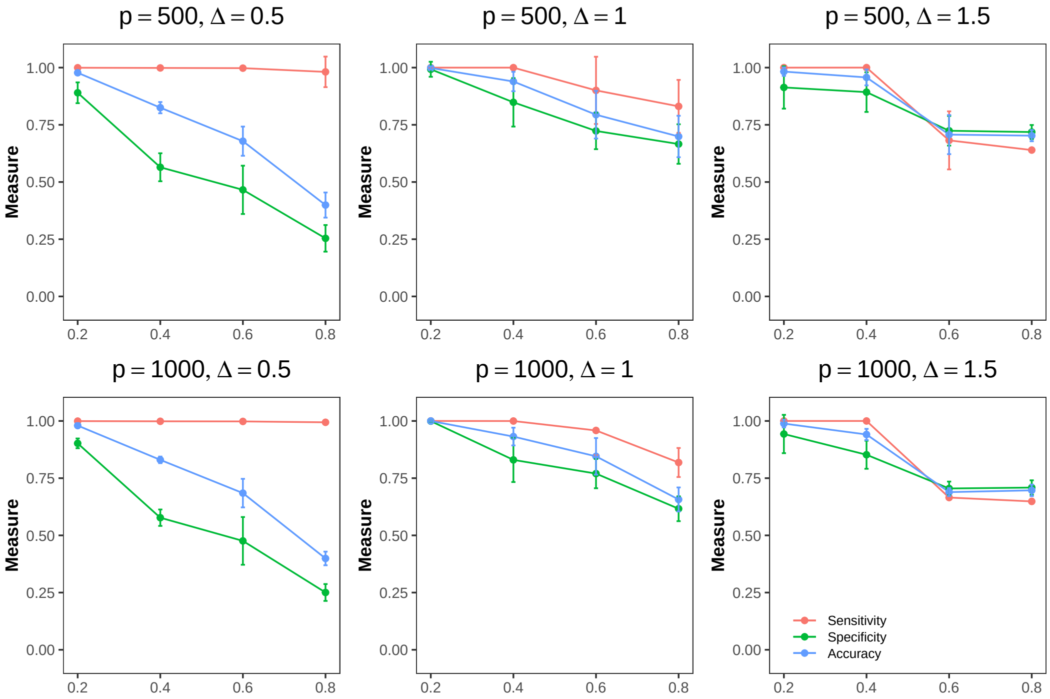

Figure S5: Simulation study: DGs selection performance of BISON across the various simulated scenarios, in terms of different metrics. Each subplot represents a specific combination of signal strength (Δ) and the number of genes (p). The x-axis denotes the expected proportion of nonDGs (π_0), while the different lines correspond to the different metrics, specifically sensitivity (red), specificity (green), and accuracy (blue). For each scenario, the mean (point) and standard deviation (interval) of each metric, computed across the 50 generated datasets, are provided.

S2.6 Model selection

Figures S6 and S7 show the performance in terms of the average estimated number of spot and gene clusters, respectively. Each subplot represents a specific combination of signal strength (Δ) and the number of genes (p), while the x-axis denotes the expected proportion of nonDGs (π_0). Overall, the performance in terms of the number of spot clusters is robust. However, it shows limitations when the number of genes is small ($p = 500$), the signal strength is weak ($\Delta = 0.5$), and the expected proportion of nonDGs is high ($\pi_0 = 0.8$). As for performance in terms of the number of gene clusters, the results are similar and show only limitations when $\pi_0 = 0.8$.

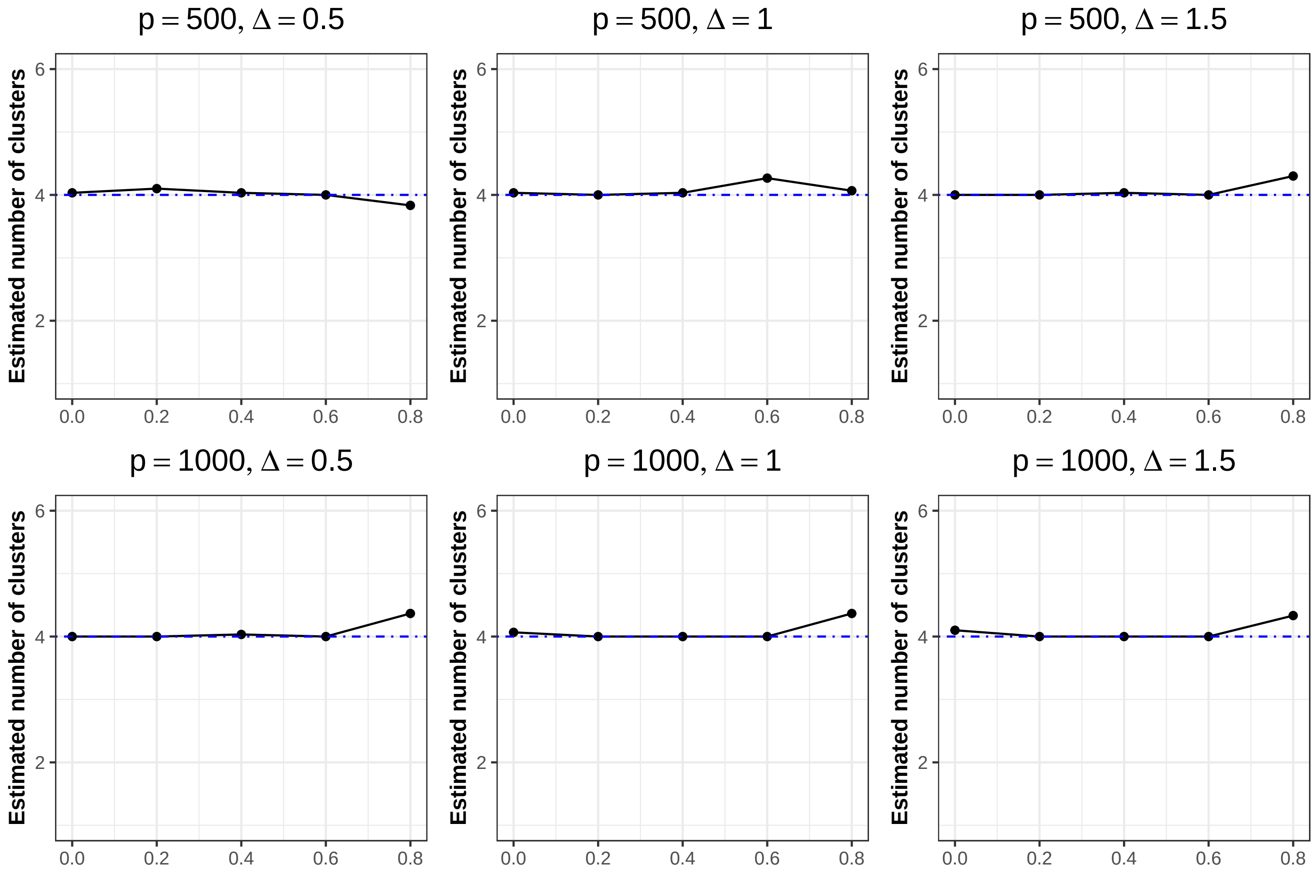

Figure S6: Simulation study: Estimated number of clusters for spots clustering against the expected proportion of nonDGs π_0. Each subplot represents a specific combination of signal strength (Δ) and the number of genes (p), as indicated on top of the subplot. Horizontal blue dash and point line indicate the value used to simulate the data (benchmark).

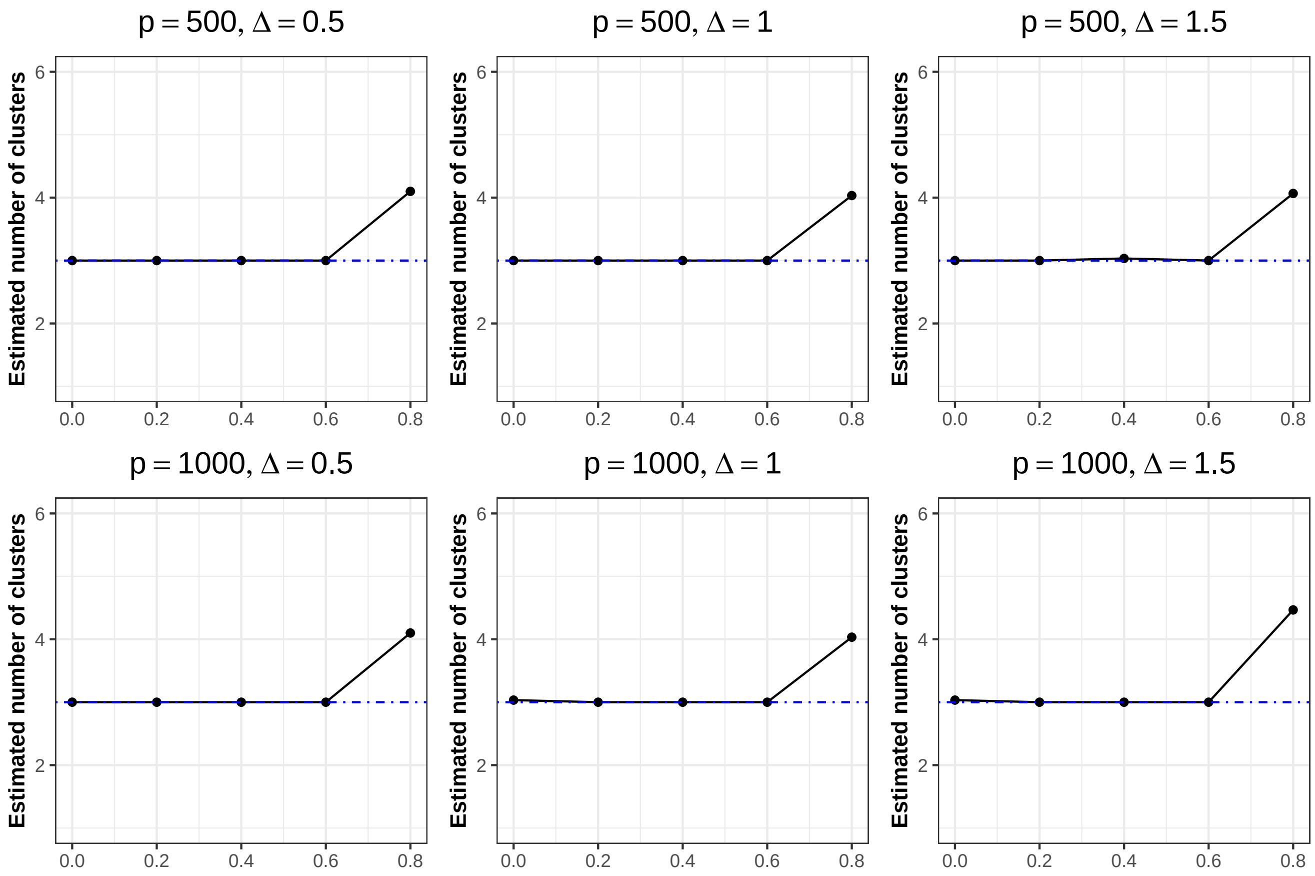

Figure S7: Simulation study: Estimated number of clusters for genes clustering against the expected proportion of nonDGs π_0. Each subplot represents a specific combination of signal strength (Δ) and the number of genes (p), as indicated on top of the subplot. Horizontal blue dash and point line indicates the value used to simulate the data (benchmark).

S2.7 Ablation study

To evaluate the contribution of including spatial information through the MRF prior and of incorporating feature selection in our model, we conduct an ablation study. Specifically, we implement BISON without MRF (BISON w/o MRF) by assigning the hyperparameter $f = 0$, such that no spatial information is incorporated. Further, we implement a version of BISON without feature selection (BISON w/o FS). We then fit these reduced models on the simulated data of Section 3 of the manuscript. Figure S8 depicts the spot clustering performance of BISON w/o MRF and BISON w/o FS, where BISON consistently achieves the best performance. Figure S9 shows the gene clustering performance of BISON w/o MRF and BISON w/o FS. BISON in almost all cases shows superior performance, especially for lower signals. These results indicate that it is important to incorporate the MRF prior and feature selection in our model.

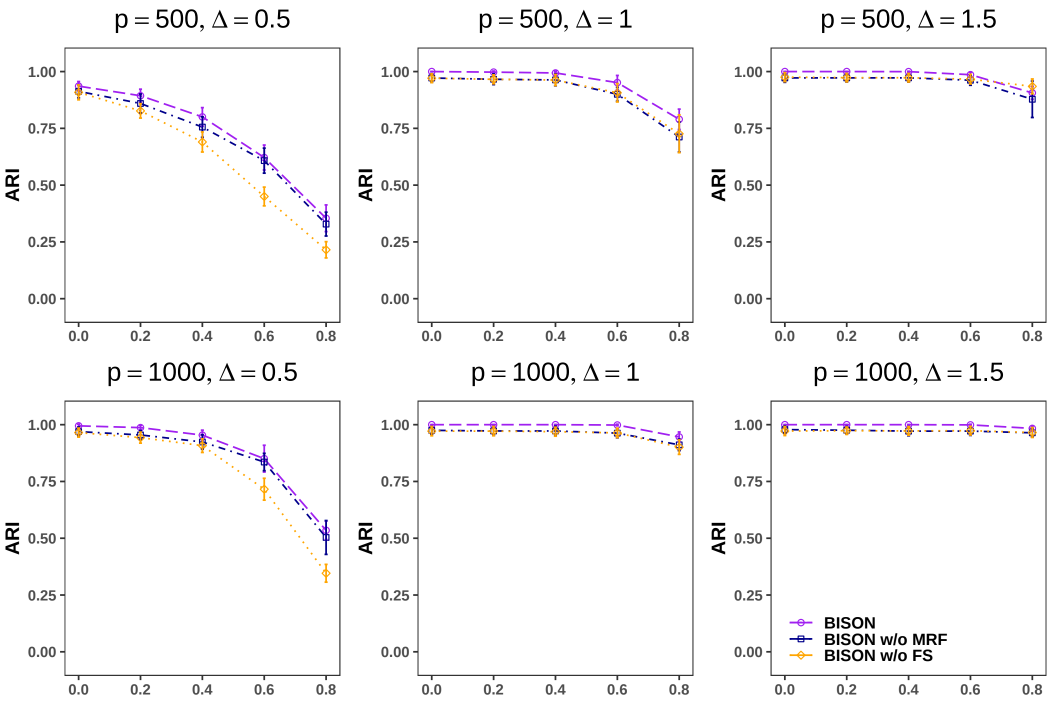

Figure S8: Ablation study on simulated data: The Adjusted Rand Index (ARI) for spot clustering against the expected proportion of nonDGs (π_0). Each subplot represents a specific combination of signal strength (Δ) and number of genes (p), as indicated at the top of each subplot. For each scenario, the mean (point) and standard deviation (interval) of ARI are computed across 50 generated datasets.

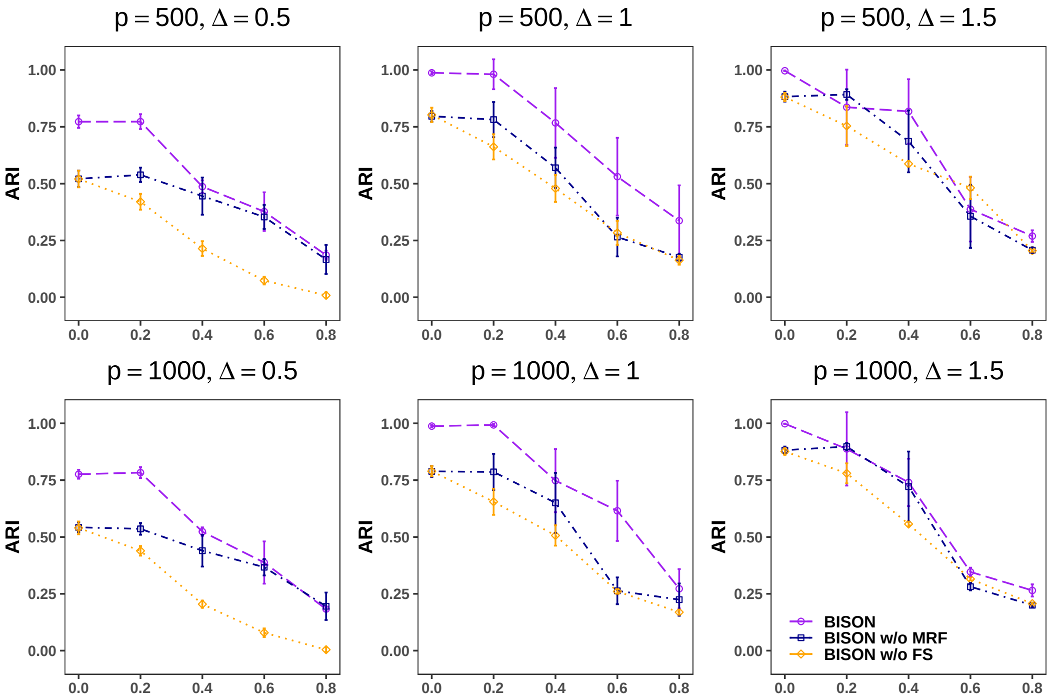

Figure S9: Ablation study on simulated data: The Adjusted Rand Index (ARI) for gene clustering against the expected proportion of nonDGs (π_0). Each subplot represents a specific combination of signal strength (Δ) and number of genes (p), as indicated at the top of each subplot. For each scenario, the mean (point) and standard deviation (interval) of the ARI are computed across 50 generated datasets.

S3 Applications

In this section, we present additional tables and figures related to the case study. Figure S10 illustrates the lattice structure of SRT data. On the left, the spots in SRT data from the ST platform are arranged in a square lattice, where each spot has at most four neighbors. On the right, the spots in SRT data from the 10x Visium platform are arranged in a triangular lattice, where each spot has up to six neighbors.

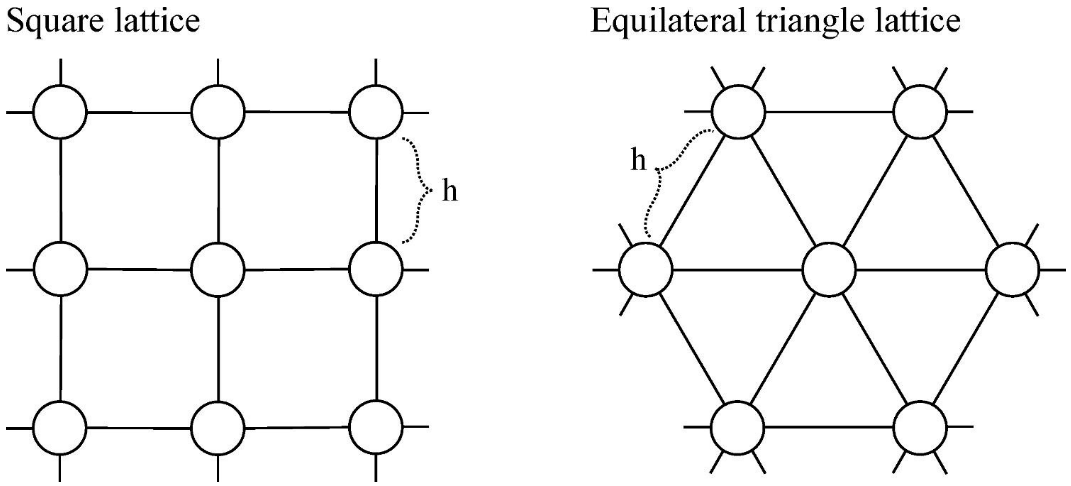

Figure S10: Lattice structure of SRT data from the ST (left) and Visium (right) platforms.

Figure S11 shows the hierarchical clustering results of MOB ST data for DGs identified by BNPSpace. The identified discriminating genes are grouped into three distinct gene clusters.

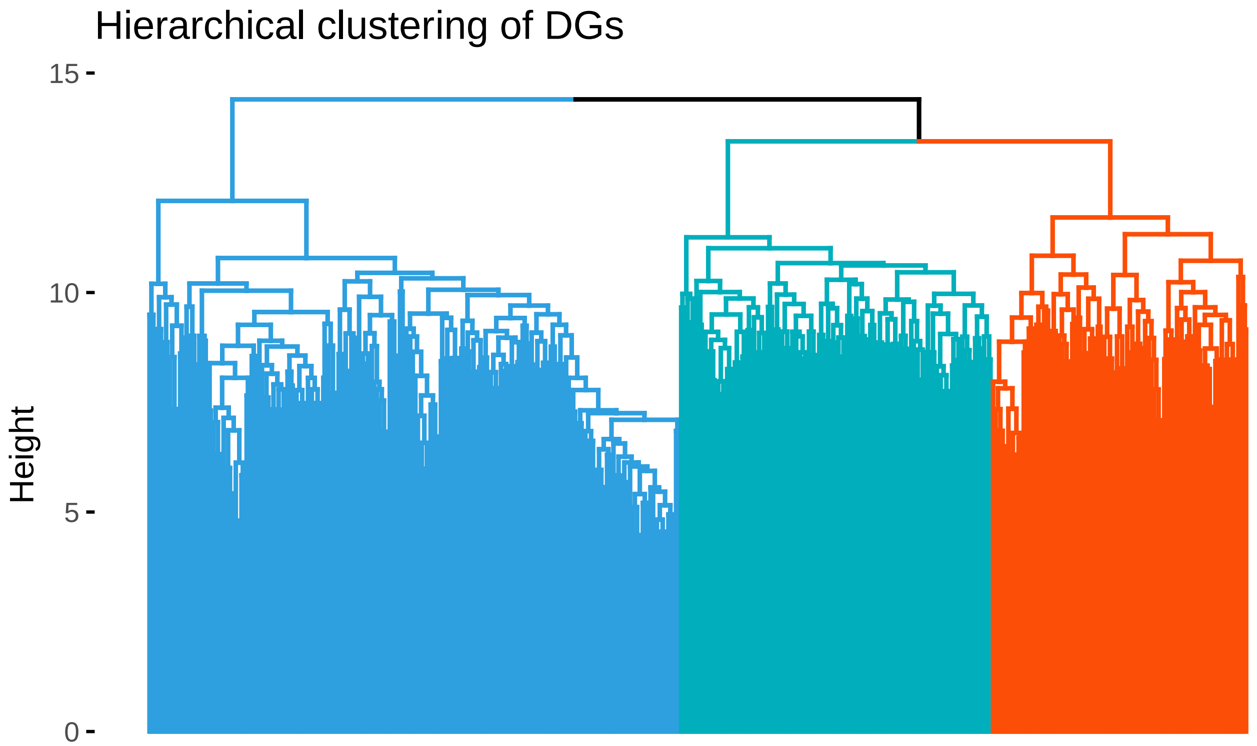

Figure S11: MOB ST data: Hierarchical clustering results for DGs identified by BNPSpace. The identified discriminating genes are grouped into three distinct gene clusters.

Figure S12 presents the trace plots of the posterior samples for π_0, μ_{11}, μ_{12}, μ_{21}, and μ_{22} in the MOB ST data. Each trace plot is displayed as a subfigure, with different colors representing one of the three MCMC chains that were run. The trace plots indicate clear convergence after a few iterations.

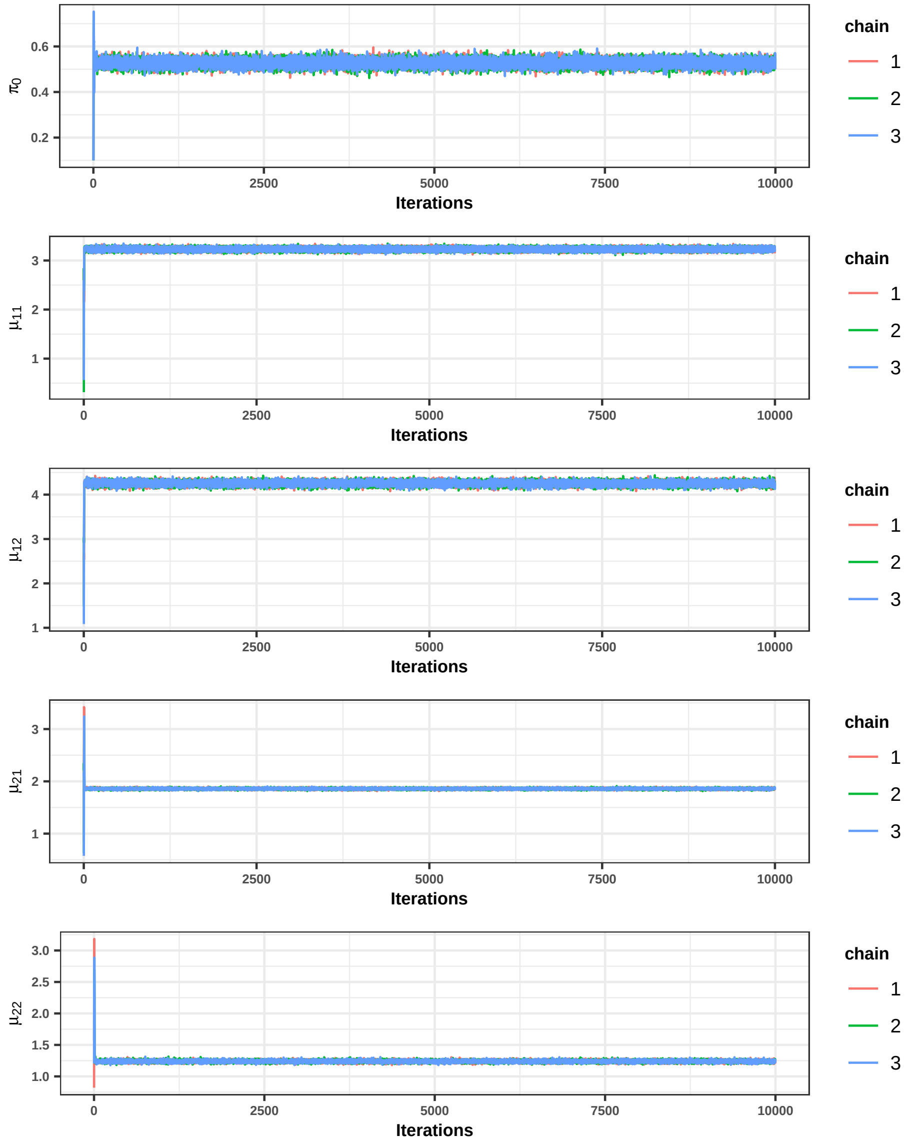

Figure S12: MOB ST data: Trace plots of the posterior samples for π_0, μ_{11}, μ_{12}, μ_{21}, and μ_{22}. Each trace plot is displayed as a subfigure, with different colors representing one of the three MCMC chains that were run.

Figure S13 presents the trace plots of the posterior samples for π_0, μ_{11}, μ_{12}, μ_{21}, and μ_{22} in the HBC 10x Visium data. Each trace plot is displayed as a subfigure, with different colors representing one of the three MCMC chains that were run. The trace plots indicate clear convergence after a few iterations.

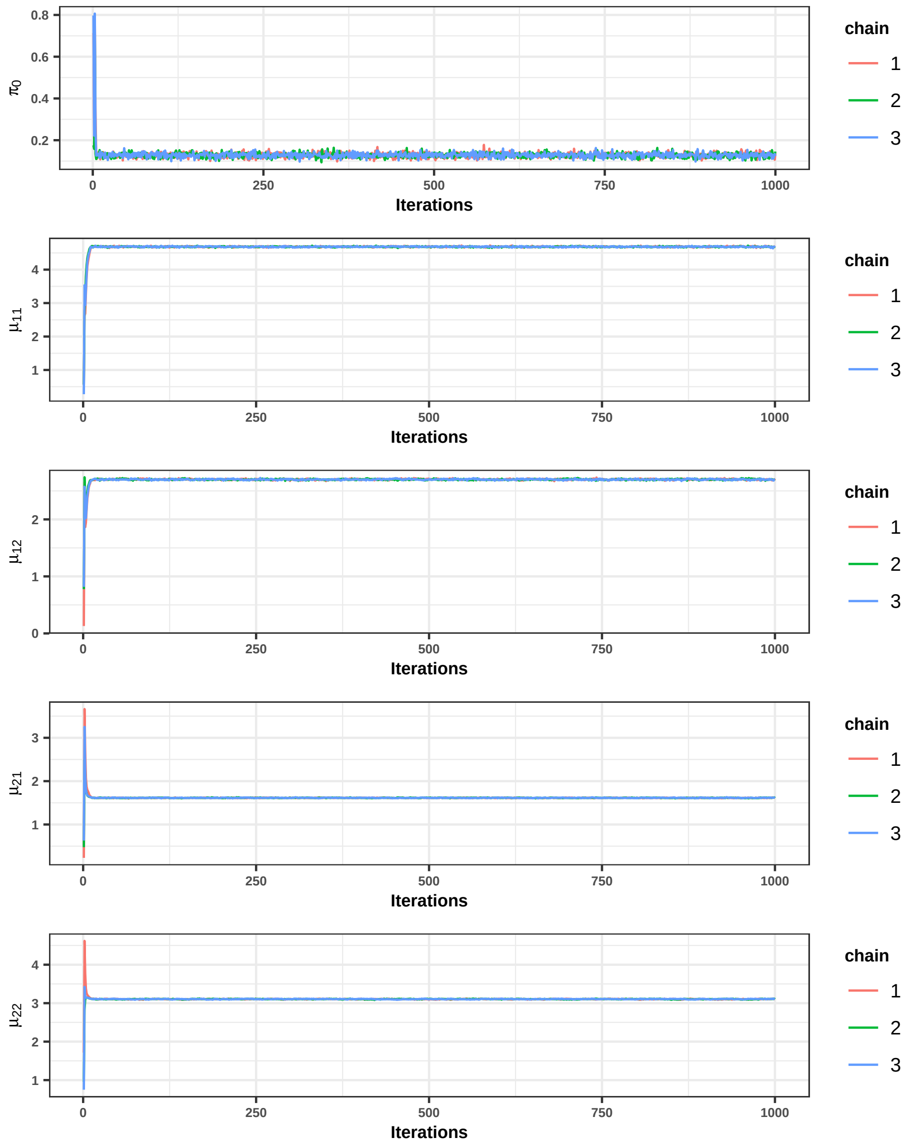

Figure S13: HBC 10x Visium data: Trace plots of the posterior samples for π_0, μ_{11}, μ_{12}, μ_{21}, and μ_{22}. Each trace plot is displayed as a subfigure, with different colors representing one of the three MCMC chains that were run.

Figure S14 presents the trace plots of the posterior samples for π_0, μ_{11}, μ_{12}, μ_{21}, and μ_{22} in the mPFC STARmap data. Each trace plot is displayed as a subfigure, with different colors representing one of the three MCMC chains that were run. The trace plots indicate clear convergence after a few iterations.

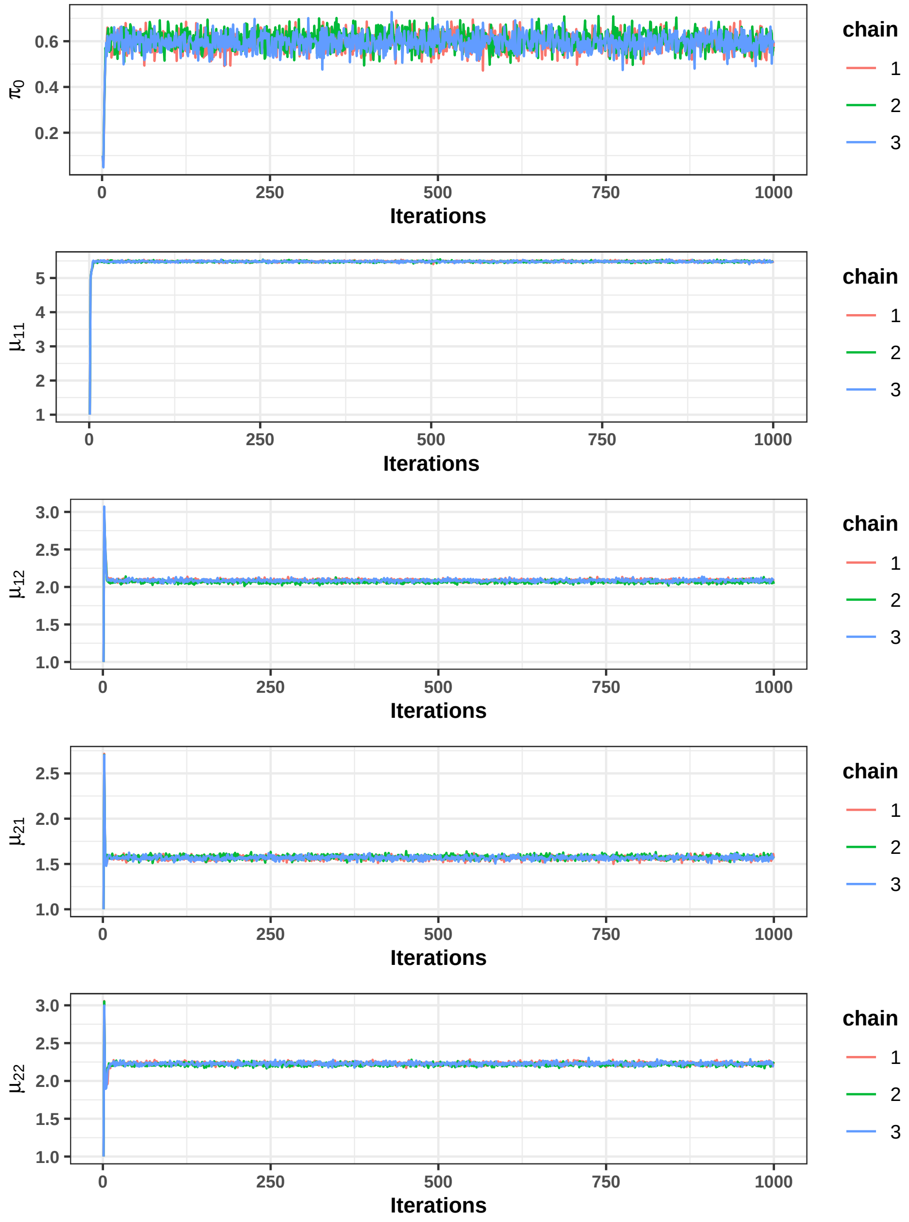

Figure S14: mPFC STARmap data: Trace plots of the posterior samples for π_0, μ_{11}, μ_{12}, μ_{21}, and μ_{22}. Each trace plot is displayed as a subfigure, with different colors representing one of the three MCMC chains that were run.

Table S1 provides the top five mICL values in the range considered for the MOB ST data, with the optimal model corresponding to $K = 4$ and $R = 3$.

Table S1: MOB ST data: top five mICL values.

K	R	mICL	ARI
4	3	484455	0.537
4	2	485210	0.531
4	4	485439	0.532
4	6	485479	0.524
5	3	485814	0.512

Table S2 provides the top five mICL values in the range considered for the HBC 10x Visium data, with the optimal model corresponding to $K = 5$ and $R = 4$.

Table S2: HBC 10x Visium data: top five mICL values.

K	R	mICL	ARI
5	4	4.932×10^6	0.487
5	5	4.939×10^6	0.479
5	6	4.952×10^6	0.481
5	5	4.999×10^6	0.451
4	3	5.037×10^6	0.462

Table S3 provides the top five mICL values in the range considered for the mPFC STARmap data, with the optimal model corresponding to $K = 3$ and $L = 3$.

Table S3: mPFC STARmap data: top five mICL values.

K	R	mICL	ARI
3	3	7.171×10^5	0.591
3	2	7.172×10^5	0.597
3	4	7.212×10^5	0.565
4	3	7.219×10^5	0.582
4	2	7.237×10^5	0.566

Figure S15 shows the manual spatial domain annotation (benchmark) for the MOB ST data

and the domain identification results for BISON and several representative spatial domain identification methods, including Louvain, BayesSpace (Zhao et al. 2021), SpaGCN (Hu et al. 2021), and STAGATE (Dong & Zhang 2022).

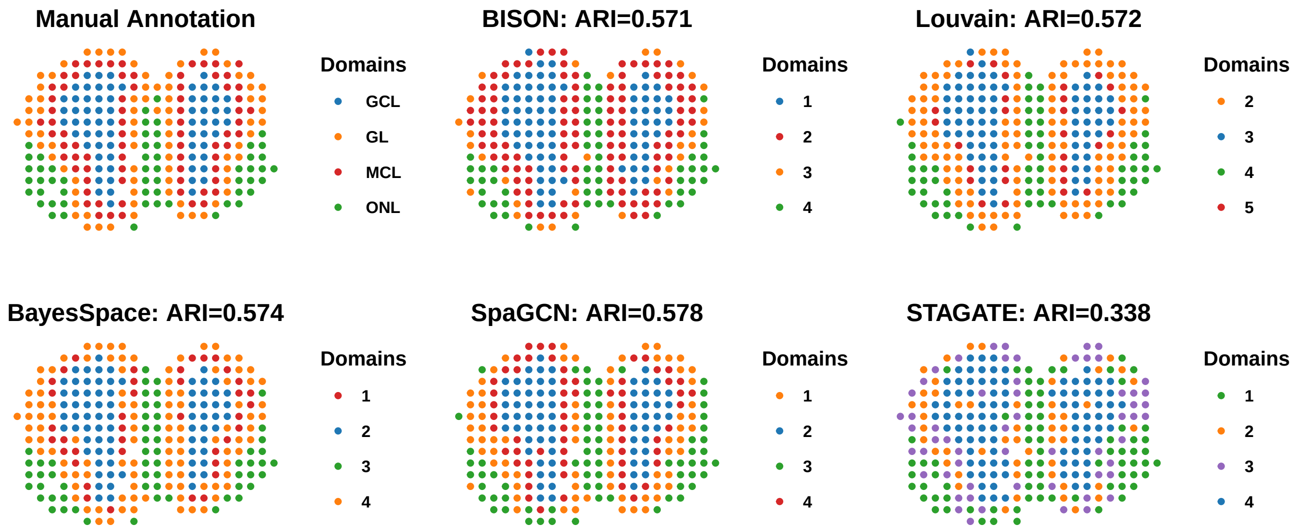

Figure S15: MOB ST data: Manual spatial domain annotation (benchmark) and spatial domain identification of BISON and competing methods.

Figure S16 shows the manual spatial domain annotation (benchmark) for the HBC 10x Visium data and the domain identification results for BISON and several representative spatial domain identification methods, including Louvain, BayesSpace, SpaGCN, and STAGATE.

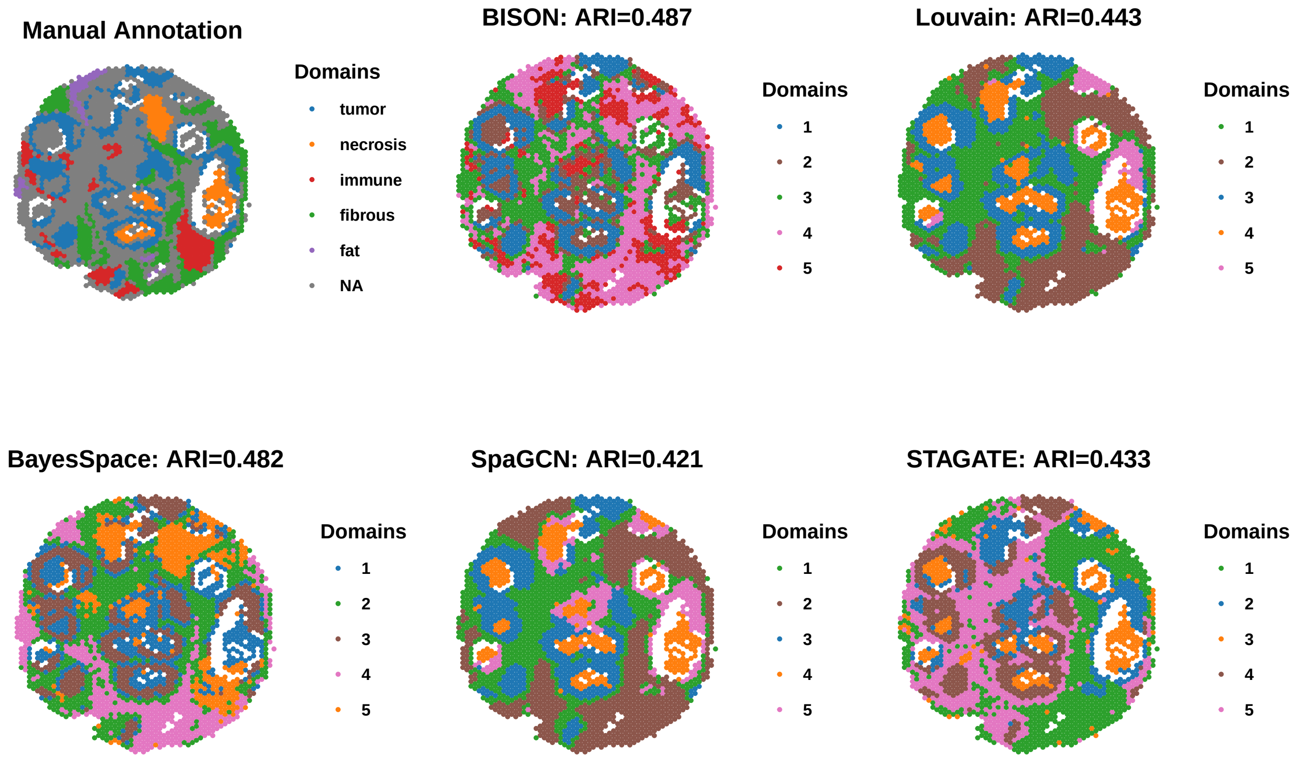

Figure S16: HBC 10x Visium data: Manual spatial domain annotation (benchmark) and spatial domain identification of BISON and competing methods.

Figure S17 shows the manual spatial domain annotation (benchmark) for the mPFC STARmap data and the domain identification results for BISON and several representative spatial domain identification methods, including Louvain, BayesSpace, SpaGCN, and STAGATE.

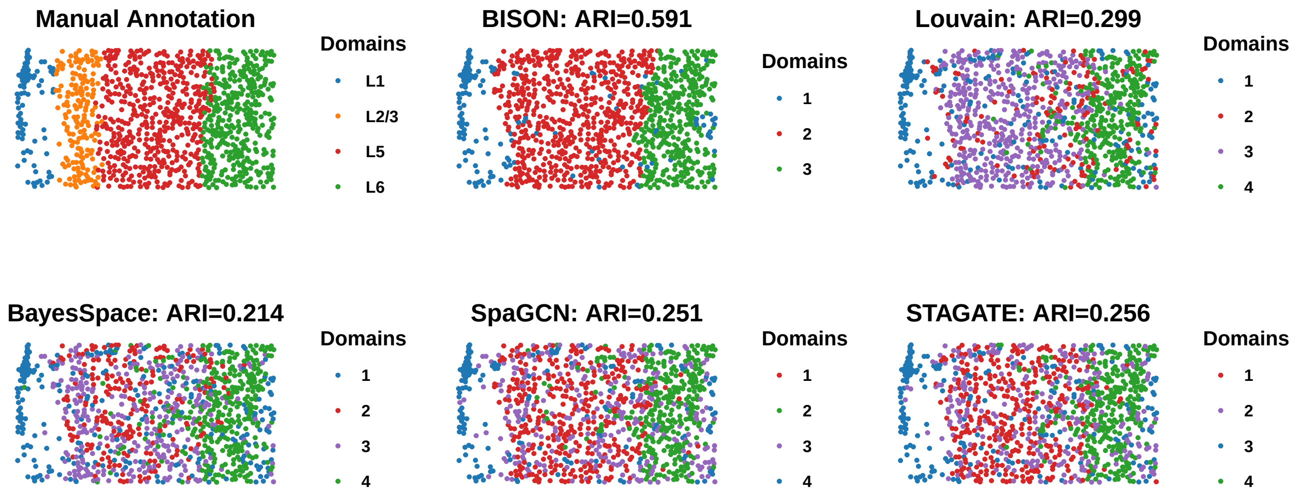

Figure S17: mPFC STARmap data: Manual spatial domain annotation (benchmark) and spatial domain identification of BISON and competing methods.

Figure S18 displays the results of a GO enrichment analysis of biological processes (BP) for gene groups identified by BISON in the HBC 10x Visium data analysis. Figure S19 displays the results of a GO enrichment analysis of biological processes (BP) for gene groups identified by BISON in the mPFC STARmap data analysis.

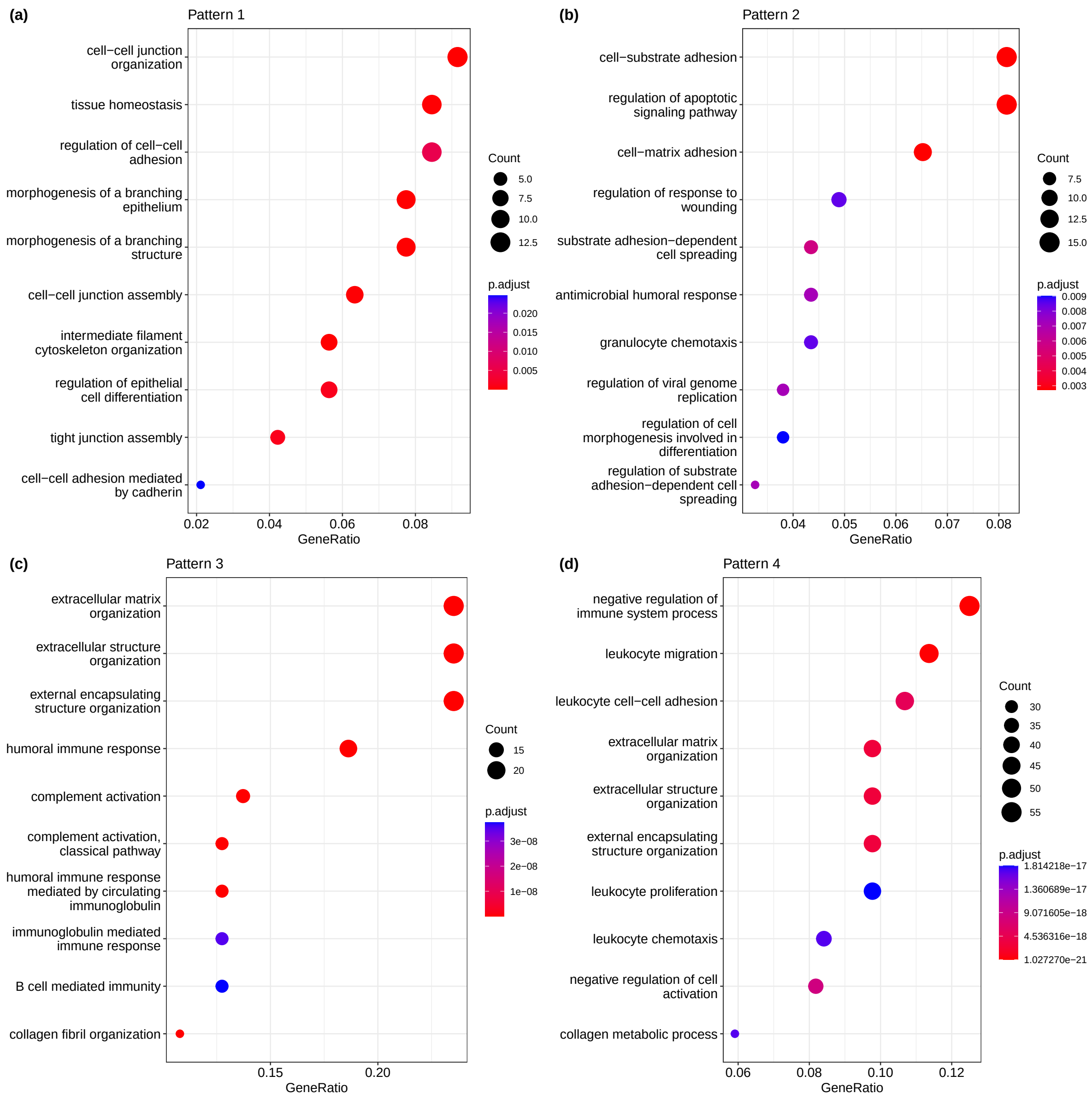

Figure S18: HBC 10x Visium data: GO enrichment analysis of biological process for the four informative gene groups identified by BISON. The x-axis represents the gene ratio in each enriched pathway.

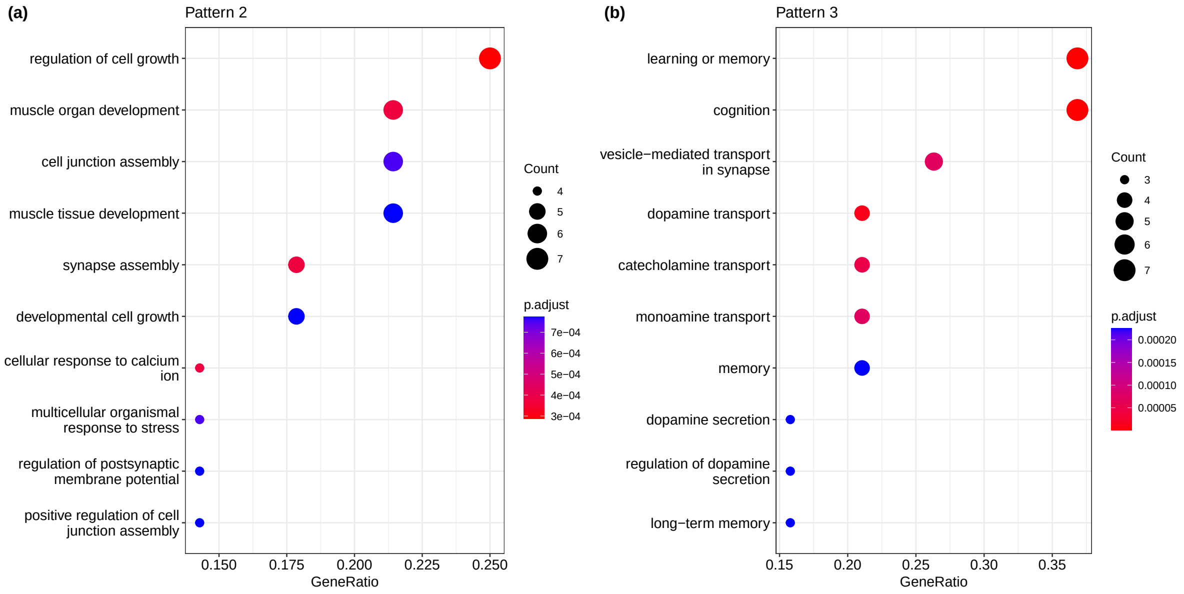

Figure S19: mPFC STARmap data: GO enrichment analysis of biological process for genes in Patterns 2 and 3. The x-axis represents the gene ratio in each enriched pathway.

Table S4: Computation time (in seconds) of all methods across different simulation patterns and real datasets. Computation is performed on a single thread of an E5-2643 v4 CPU (20 MB cache, 3.40 GHz) with 256 GB of memory. For simulations, the reported time is the mean execution time over 50 replications. For BISON, the reported time corresponds to 100 iterations. SpaRTaCo fails to converge when applied to the mPFC STARmap dataset.

**Dataset**	n	p	**BISON**	**SpaRTaCo**	**BC**	**SparseBC**	K**-means**
Simulated data	274	500	52	136	0.92	0.91	0.14
	274	1000	109	192	1.82	1.81	0.24
MOB ST data	274	1000	115	203	1.81	1.78	0.23
HBC 10x Visium data	2518	1000	2762	3221	20	21	3.1
mPFC STARmap data	1049	166	121	-	1.68	1.76	0.31

References

Dong, K. & Zhang, S. (2022), Deciphering spatial domains from spatially resolved transcriptomics with an adaptive graph attention auto-encoder, *Nat. Commun.* **13**(1), 1739.

Hu, J. et al. (2021), SpaGCN: Integrating gene expression, spatial location and histology to identify spatial domains and spatially variable genes by graph convolutional network, *Nat. Methods* **18**(11), 1342–1351.

Hubert, L. & Arabie, P. (1985), Comparing partitions, *J. Classif.* **2**, 193–218.

Sottosanti, A. & Risso, D. (2023), Co-clustering of spatially resolved transcriptomic data, *Ann. Appl. Stat.* **17**(2), 1444.

Zhao, E. et al. (2021), Spatial transcriptomics at subspot resolution with BayesSpace, *Nat. Biotechnol.* **39**(11), 1375–1384.
